# Supplementary material for: Global-, Regional-, and National-Level Impacts of the COVID-19 Pandemic on Tuberculosis Diagnoses, 2020–2021
Source: Microorganisms. 2023 Aug 30;11(9):2191. doi: 10.3390/microorganisms11092191 (PMC10536333; doi:10.3390/microorganisms11092191)
Supplement: Supplementary file 1 [file microorganisms-11-02191-s001.zip › microorganisms-2557505-supplementary.pdf]

Supplementary Appendix to “Global-, regional-, and national-level impacts of the COVID-19 pandemic on tuberculosis diagnoses, 2020-2021”

Table of Contents

Supplemental figures 2

Figure S1. Geographies within each super-region from the Global Burden of Diseases, Injuries, and Risk Factors (GBD) Study 2

Figure S2. Temporal trends in observed and expected tuberculosis notification rates by Global Burden of Disease super-regions, 2013-2021 3

Figure S3. Temporal trends in observed and expected tuberculosis notification rates for the top 20 high tuberculosis burden countries in the Global Burden of Disease 2019 study, 2013-2021 4

Figure S4. Bivariate associations [unstandardized coefficients] between tuberculosis risk factors, health system, socio-demographic, public health and social measures, and COVID-19 factors on observed to expected ratios of tuberculosis diagnoses 5

Supplemental tables 6

Table S1. Difference in observed to expected tuberculosis diagnoses during the COVID-19 pandemic for 170 countries in 2020 and 2021 6

Table S2. Difference in observed to expected tuberculosis diagnoses during the COVID-19 pandemic for 65 ≥ years age group and under 65 age group in 2020 and 2021 for 155 countries 10

Table S3. Bivariate linear regression results for observed to expected ratios of tuberculosis diagnoses during the COVID-19 pandemic in 2020 20

Table S4. Bivariate linear regression results for observed to expected ratios of tuberculosis diagnoses during the COVID-19 pandemic in 2021 22

Figure S1. Geographies within each super-region from the Global Burden of Diseases, Injuries, and Risk Factors (GBD) Study

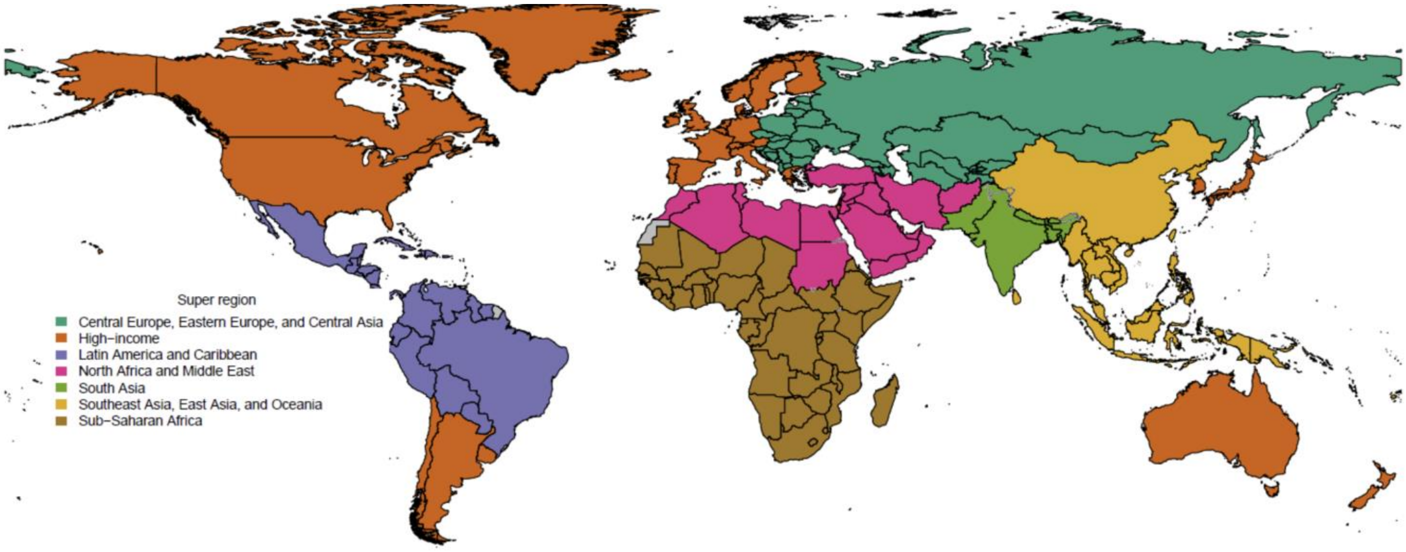

Figure S2. Temporal trends in observed and expected tuberculosis notification rates by Global Burden of Disease super-regions, 2013-2021

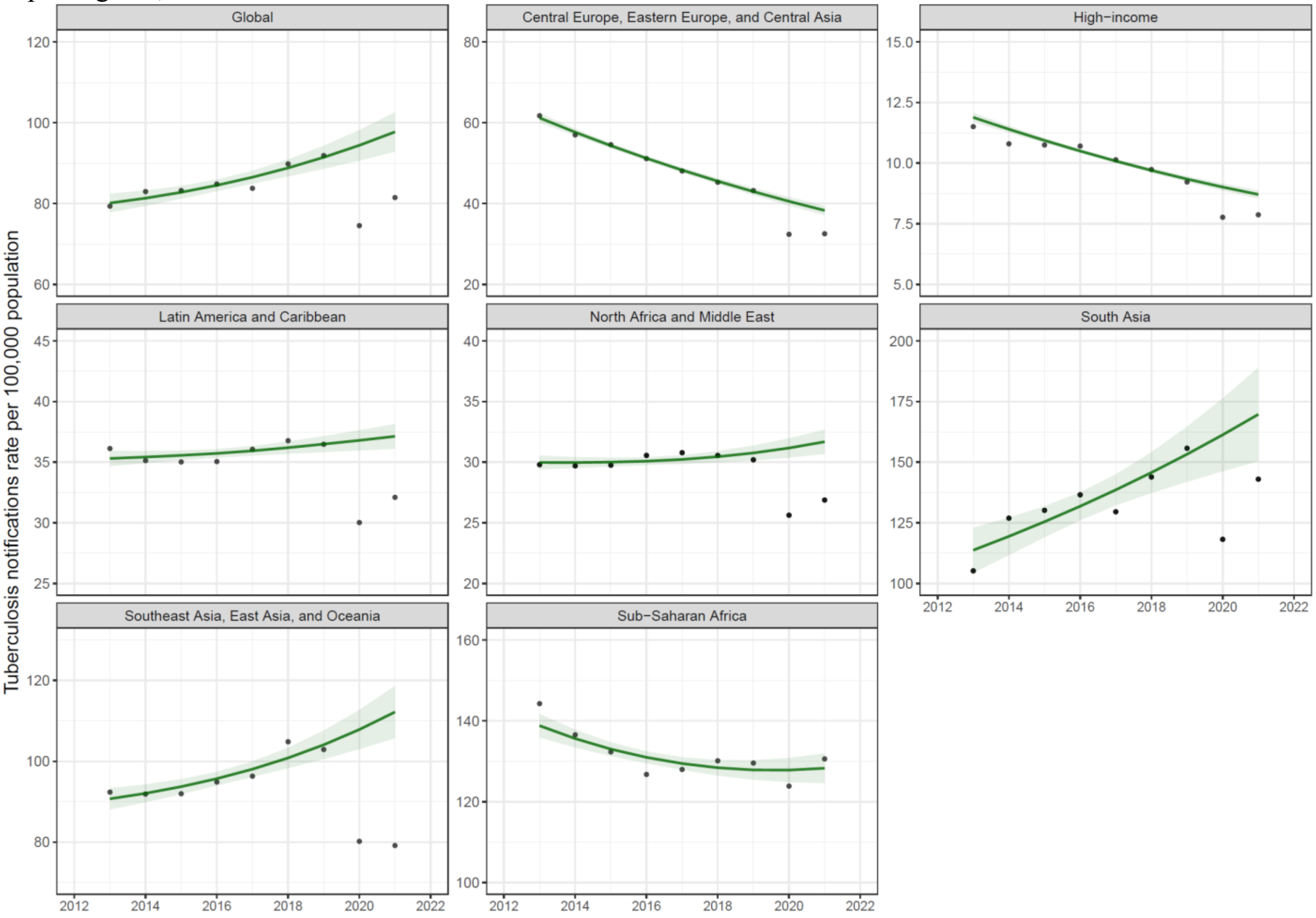

Note: The dark green represents the expected tuberculosis notification rate. The black points are the observed tuberculosis notification rate.

Figure S3. Temporal trends in observed and expected tuberculosis notification rates for the top 20 high tuberculosis burden countries in the Global Burden of Disease 2019 study, 2013-2021

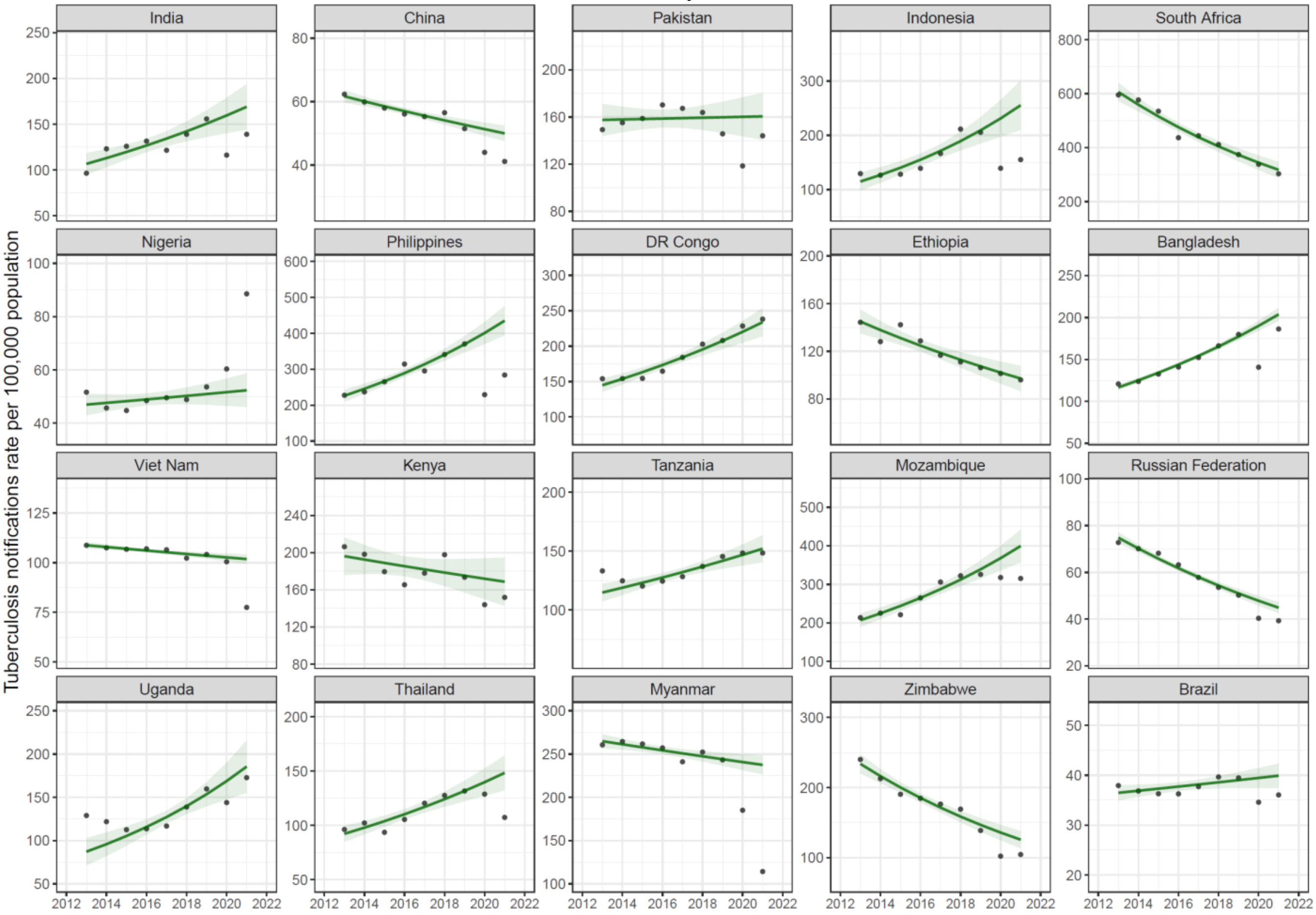

Note: These 20 countries were estimated to rank in the top 20 in terms of all-form incidence counts in 2019 based on GBD 2019 estimates. The dark green represents the expected tuberculosis notification rate. The black points are the observed tuberculosis notification rate.

Figure S4. Bivariate associations [unstandardized coefficients] between tuberculosis risk factors, health system, socio-demographic, public health and social measures, and COVID-19 factors on observed to expected ratios of tuberculosis diagnoses

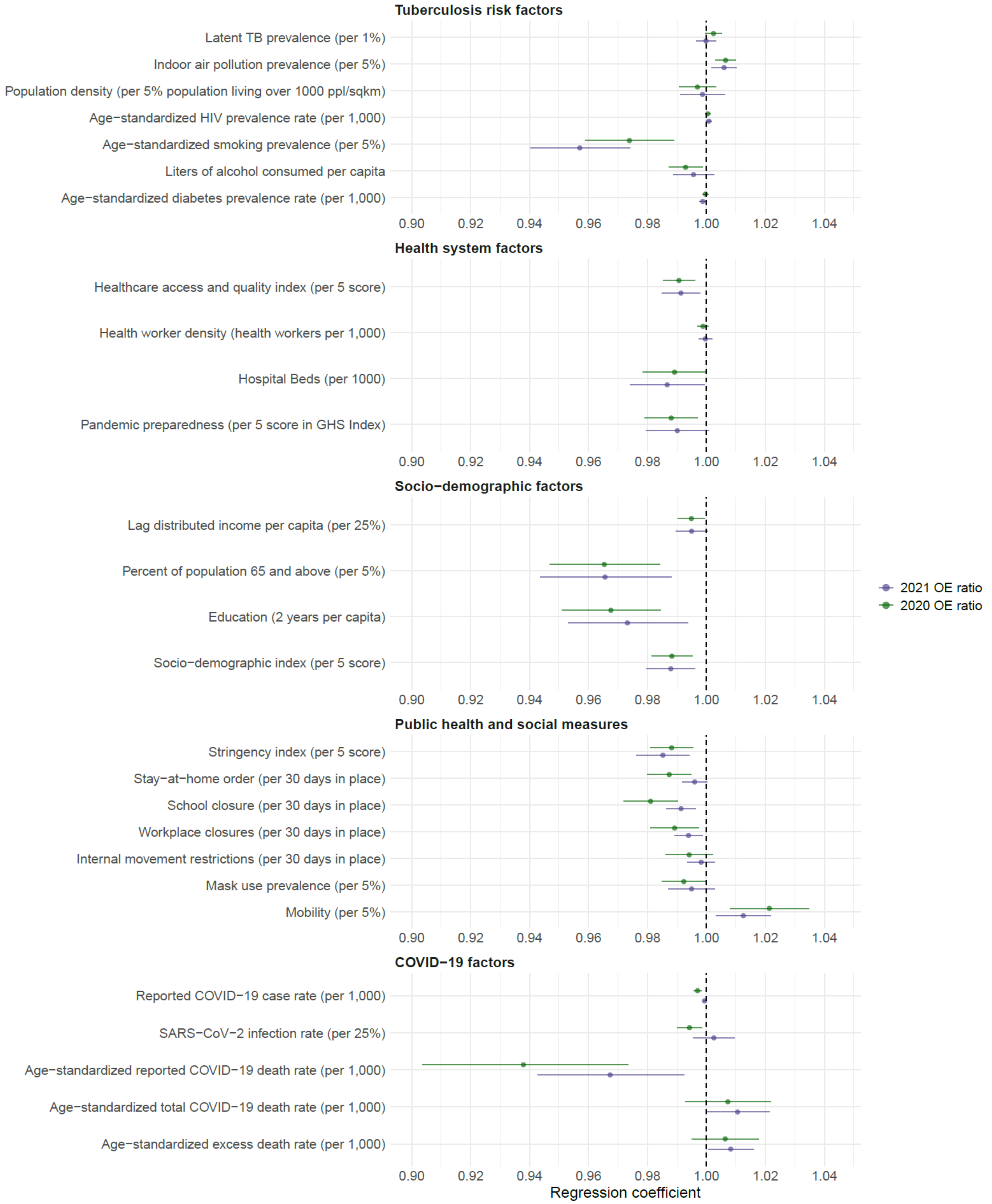

Table S1. Difference in observed to expected tuberculosis diagnoses during the COVID-19 pandemic for 170 countries in 2020 and 2021.

		Observed TB notifications		Expected TB notifications		Difference in observed to expected TB notifications	
Location	Year	Cases	Rate	Cases	Rate	Number	Ratio
Global	2020	5830000	74.5	7380000 (7090000, 7680000)	94.4 (90.6, 98.2)	-1550000 (-1850000, -1260000)	0.79 (0.75, 0.83)
	2021	6430000	81.5	7710000 (7330000, 8100000)	97.7 (92.8, 102.6)	-1280000 (-1670000, -897000)	0.83 (0.79, 0.88)
Southeast Asia, East Asia, and Oceania	2020	1740000	80.2	2350000 (2240000, 2450000)	107.8 (103, 112.7)	-601000 (-706000, -496000)	0.74 (0.70, 0.78)
	2021	1730000	79.2	2450000 (2310000, 2590000)	112.2 (105.7, 118.6)	-721000 (-863000, -580000)	0.71 (0.66, 0.75)
East Asia	2020	714000	48.6	824000 (792000, 856000)	56.1 (53.9, 58.3)	-110000 (-142000, -77100)	0.87 (0.83, 0.9)
	2021	673000	45.7	805000 (767000, 843000)	54.7 (52.1, 57.3)	-132000 (-171000, -94200)	0.84 (0.79, 0.88)
China	2020	625000	44	729000 (699000, 758000)	51.3 (49.2, 53.4)	-104000 (-133000, -74300)	0.86 (0.82, 0.9)
	2021	585000	41.1	711000 (677000, 746000)	50 (47.6, 52.4)	-126000 (-161000, -91300)	0.82 (0.78, 0.87)
Democratic People's Republic of Korea	2020	89600	340.3	95400 (82000, 109000)	362.1 (311.2, 413.1)	-5750 (-19200, 7670)	0.94 (0.8, 1.08)
	2021	87400	331.2	93800 (77900, 110000)	355.3 (295.2, 415.3)	-6350 (-22200, 9510)	0.93 (0.77, 1.1)
Southeast Asia	2020	1000000	144.6	1490000 (1390000, 1590000)	215.2 (200.8, 229.7)	-489000 (-589000, -389000)	0.67 (0.62, 0.73)
	2021	1030000	147	1610000 (1480000, 1750000)	230.9 (211.4, 250.3)	-586000 (-721000, -450000)	0.64 (0.57, 0.7)
Cambodia	2020	29000	172.6	27600 (23800, 31400)	163.9 (141.3, 186.4)	1470 (-2340, 5280)	1.05 (0.91, 1.19)
	2021	21600	126.7	26000 (21800, 30300)	152.8 (127.6, 178)	-4450 (-8750, -156)	0.83 (0.68, 0.98)
Indonesia	2020	384000	139.2	637000 (544000, 730000)	231 (197.2, 264.7)	-253000 (-346000, -160000)	0.60 (0.49, 0.72)
	2021	433000	155.1	712000 (585000, 839000)	255.2 (209.7, 300.8)	-279000 (-406000, -152000)	0.61 (0.47, 0.75)
Lao People's Democratic Republic	2020	8010	110.1	7530 (7070, 8000)	103.5 (97.1, 109.9)	478 (-17.7, 974)	1.06 (1.00, 1.13)
	2021	6170	83.6	8260 (7640, 8870)	111.9 (103.5, 120.3)	-2080 (-2720, -1450)	0.75 (0.68, 0.82)
Malaysia	2020	23000	72.8	26100 (25300, 26800)	82.7 (80.4, 85.1)	-3120 (-3930, -2310)	0.88 (0.85, 0.91)
	2021	21200	66.6	26400 (25500, 27400)	83.1 (80.2, 86)	-5250 (-6210, -4290)	0.80 (0.77, 0.83)
Maldives	2020	107	21.3	159 (127, 191)	31.7 (25.3, 38)	-52.2 (-89.8, -14.5)	0.67 (0.45, 0.9)
	2021	87	16.8	163 (124, 203)	31.6 (24, 39.2)	-76.2 (-120, -32.9)	0.53 (0.30, 0.77)
Myanmar	2020	103000	185	135000 (129000, 140000)	240.7 (231.5, 249.9)	-31100 (-36300, -25900)	0.77 (0.73, 0.80)
	2021	64400	114.2	134000 (128000, 140000)	237.4 (226.5, 248.4)	-69600 (-75700, -63400)	0.48 (0.45, 0.51)
Philippines	2020	257000	229.2	449000 (414000, 483000)	400.9 (370.2, 431.7)	-192000 (-227000, -158000)	0.57 (0.51, 0.63)
	2021	322000	283.9	493000 (447000, 539000)	435.1 (394.5, 475.7)	-171000 (-217000, -125000)	0.65 (0.58, 0.73)
Sri Lanka	2020	7040	31.8	8020 (7680, 8360)	36.2 (34.7, 37.7)	-981 (-1360, -603)	0.88 (0.83, 0.92)
	2021	6550	29.4	7820 (7420, 8220)	35.1 (33.3, 36.9)	-1270 (-1700, -838)	0.84 (0.79, 0.89)
Thailand	2020	85800	128.6	93200 (84800, 102000)	139.7 (127.1, 152.2)	-7360 (-15700, 1010)	0.92 (0.83, 1.01)
	2021	71500	107.2	98800 (88100, 110000)	148.2 (132.1, 164.3)	-27400 (-38100, -16600)	0.72 (0.63, 0.82)
Timor-Leste	2020	3220	235.5	3850 (3450, 4240)	280.9 (252.1, 309.8)	-622 (-1030, -211)	0.84 (0.74, 0.94)
	2021	3190	228.4	3890 (3410, 4370)	278.5 (244, 313)	-699 (-1190, -205)	0.82 (0.70, 0.94)
Viet Nam	2020	99900	100.5	102000 (1e+05, 104000)	102.6 (100.7, 104.6)	-2160 (-4220, -105)	0.98 (0.96, 1.00)
	2021	77700	77.4	102000 (99700, 104000)	101.8 (99.4, 104.2)	-24400 (-26800, -22000)	0.76 (0.74, 0.78)
Oceania	2020	29700	218.2	32500 (30200, 34900)	239.1 (221.6, 256.5)	-2850 (-5250, -449)	0.91 (0.84, 0.98)
	2021	30200	216.6	33700 (30700, 36600)	241.6 (220.3, 263)	-3480 (-6470, -487)	0.90 (0.81, 0.98)

Fiji	2020	431	46.8	464 (366, 563)	50.4 (39.7, 61.1)	-33.4 (-140, 73.4)	0.93 (0.70, 1.15)
	2021	342	37	495 (367, 623)	53.6 (39.7, 67.4)	-153 (-286, -20.4)	0.69 (0.46, 0.92)
Kiribati	2020	385	321.7	383 (277, 488)	319.8 (231.7, 407.9)	2.29 (-110, 115)	1.01 (0.71, 1.30)
	2021	330	272.4	374 (250, 497)	308.4 (206.3, 410.4)	-43.6 (-172, 85.1)	0.88 (0.56, 1.21)
Marshall Islands	2020	147	262	249 (209, 290)	444.4 (372.6, 516.3)	-102 (-149, -55.6)	0.59 (0.41, 0.77)
	2021	118	209.7	272 (219, 325)	483.8 (389.6, 578)	-154 (-211, -97.1)	0.43 (0.26, 0.61)
Micronesia (Federated States of)	2020	92	89.6	83.7 (48.8, 119)	81.5 (47.5, 115.4)	8.3 (-31.3, 47.9)	1.1 (0.61, 1.59)
	2021	72	70.2	74.6 (36.6, 113)	72.7 (35.7, 109.7)	-2.59 (-44, 38.9)	0.97 (0.42, 1.51)
Papua New Guinea	2020	28200	277.4	30900 (28500, 33300)	303.6 (280.3, 326.9)	-2660 (-5060, -271)	0.91 (0.84, 0.99)
	2021	28900	276	32000 (29000, 34900)	305.7 (277.3, 334)	-3110 (-6090, -121)	0.90 (0.81, 0.99)
Solomon Islands	2020	321	47.9	380 (332, 427)	56.6 (49.5, 63.7)	-58.8 (-118, 0.344)	0.85 (0.69, 1.00)
	2021	370	54.1	380 (323, 438)	55.7 (47.3, 64.1)	-10.5 (-79.2, 58.2)	0.97 (0.79, 1.15)
Vanuatu	2020	83	27.1	83.1 (66.5, 99.7)	27.1 (21.7, 32.5)	-0.0912 (-24.5, 24.3)	1.00 (0.71, 1.29)
	2021	69	22	78.2 (59.5, 96.9)	25 (19, 31)	-9.19 (-34, 15.6)	0.88 (0.57, 1.20)
Central Europe, Eastern Europe, and Central Asia	2020	136000	32.4	170000 (165000, 174000)	40.6 (39.5, 41.6)	-34200 (-38600, -29800)	0.80 (0.78, 0.82)
	2021	136000	32.5	160000 (155000, 165000)	38.3 (37.1, 39.5)	-24100 (-29000, -19100)	0.85 (0.82, 0.88)
Central Asia	2020	41600	44	50900 (48300, 53500)	53.8 (51, 56.6)	-9260 (-11900, -6600)	0.82 (0.77, 0.87)
	2021	42200	44.1	49000 (46000, 51900)	51.1 (48, 54.2)	-6750 (-9770, -3730)	0.86 (0.80, 0.92)
Armenia	2020	394	13.1	565 (515, 616)	18.8 (17.1, 20.5)	-171 (-235, -107)	0.70 (0.59, 0.81)
	2021	398	13.3	492 (439, 544)	16.4 (14.7, 18.2)	-93.7 (-159, -28.3)	0.81 (0.68, 0.94)
Azerbaijan	2020	3680	35.3	4650 (4500, 4800)	44.6 (43.1, 46)	-970 (-1160, -779)	0.79 (0.75, 0.83)
	2021	3660	34.9	4480 (4310, 4650)	42.7 (41, 44.3)	-818 (-1030, -608)	0.82 (0.77, 0.86)
Georgia	2020	1670	45.8	2060 (1900, 2210)	56.5 (52.2, 60.7)	-387 (-562, -212)	0.81 (0.73, 0.89)
	2021	1500	41.6	1900 (1730, 2070)	52.7 (47.9, 57.5)	-400 (-588, -212)	0.79 (0.70, 0.88)
Kazakhstan	2020	9600	51.3	10700 (8890, 12500)	57.2 (47.5, 66.8)	-1100 (-2920, 725)	0.90 (0.74, 1.06)
	2021	9760	51.5	10000 (7980, 12000)	52.8 (42.1, 63.4)	-243 (-2280, 1790)	0.98 (0.77, 1.18)
Kyrgyzstan	2020	4240	62.7	6200 (5700, 6690)	91.6 (84.3, 99)	-1960 (-2470, -1440)	0.68 (0.61, 0.76)
	2021	4600	67	6070 (5490, 6650)	88.4 (79.9, 96.9)	-1470 (-2070, -875)	0.76 (0.67, 0.84)
Mongolia	2020	3860	117.7	4060 (3700, 4420)	123.8 (112.9, 134.7)	-201 (-579, 177)	0.95 (0.86, 1.04)
	2021	2710	81.2	3990 (3570, 4410)	119.6 (107, 132.3)	-1280 (-1720, -847)	0.68 (0.59, 0.77)
Tajikistan	2020	4150	41.7	5840 (5610, 6070)	58.7 (56.4, 61)	-1690 (-1950, -1430)	0.71 (0.67, 0.75)
	2021	4160	40.9	5830 (5560, 6110)	57.4 (54.7, 60.1)	-1680 (-1980, -1370)	0.71 (0.67, 0.76)
Turkmenistan	2020	1920	37.5	1800 (1470, 2130)	35.2 (28.8, 41.7)	115 (-225, 455)	1.06 (0.87, 1.26)
	2021	1880	36.4	1700 (1330, 2070)	32.9 (25.7, 40.1)	181 (-199, 561)	1.11 (0.87, 1.34)
Uzbekistan	2020	12100	36	15000 (13300, 16800)	44.6 (39.4, 49.8)	-2900 (-4650, -1140)	0.81 (0.70, 0.91)
	2021	13500	39.6	14500 (12500, 16500)	42.3 (36.4, 48.2)	-944 (-2970, 1080)	0.93 (0.80, 1.07)
Central Europe	2020	13600	11.7	19900 (19500, 20300)	17.2 (16.8, 17.5)	-6330 (-6770, -5900)	0.68 (0.66, 0.70)
	2021	14000	12.1	18600 (18200, 19100)	16.2 (15.8, 16.5)	-4670 (-5150, -4190)	0.75 (0.73, 0.77)
Albania	2020	240	8.9	434 (368, 501)	16.1 (13.7, 18.6)	-194 (-267, -121)	0.55 (0.41, 0.70)
	2021	269	10.1	433 (353, 513)	16.2 (13.2, 19.2)	-164 (-250, -77.9)	0.62 (0.45, 0.79)

Bosnia and Herzegovina	2020	357	10.7	520 (473, 568)	15.5 (14.1, 17)	-163 (-224, -103)	0.69 (0.57, 0.80)
	2021	362	11	454 (405, 503)	13.7 (12.3, 15.2)	-92 (-154, -30.4)	0.80 (0.66, 0.93)
Bulgaria	2020	887	12.9	1140 (1090, 1200)	16.6 (15.8, 17.4)	-254 (-335, -173)	0.78 (0.71, 0.85)
	2021	673	9.9	1050 (992, 1120)	15.5 (14.6, 16.4)	-381 (-460, -301)	0.64 (0.56, 0.72)
Croatia	2020	177	4.2	294 (260, 329)	6.9 (6.1, 7.8)	-117 (-161, -73.9)	0.60 (0.46, 0.75)
	2021	154	3.7	269 (231, 307)	6.4 (5.5, 7.3)	-115 (-160, -70.2)	0.57 (0.41, 0.73)
Czechia	2020	360	3.4	461 (414, 507)	4.3 (3.9, 4.8)	-101 (-160, -41.4)	0.78 (0.65, 0.91)
	2021	352	3.3	455 (400, 510)	4.3 (3.8, 4.8)	-103 (-169, -36.9)	0.77 (0.63, 0.91)
Hungary	2020	384	4	481 (424, 539)	5 (4.4, 5.6)	-97.5 (-167, -28.3)	0.80 (0.66, 0.94)
	2021	314	3.3	433 (371, 495)	4.5 (3.9, 5.2)	-119 (-190, -48.4)	0.72 (0.57, 0.88)
North Macedonia	2020	143	6.5	183 (168, 197)	8.4 (7.7, 9)	-39.7 (-67.1, -12.3)	0.78 (0.62, 0.94)
	2021	143	6.6	168 (152, 184)	7.7 (7, 8.4)	-25.1 (-53.2, 3.12)	0.85 (0.68, 1.02)
Poland	2020	3150	8.2	4690 (4520, 4850)	12.2 (11.8, 12.6)	-1540 (-1730, -1340)	0.67 (0.63, 0.71)
	2021	3450	9	4410 (4230, 4590)	11.5 (11.1, 12)	-963 (-1180, -747)	0.78 (0.73, 0.83)
Romania	2020	7190	37.6	10400 (10100, 10700)	54.2 (52.7, 55.8)	-3170 (-3510, -2830)	0.69 (0.66, 0.72)
	2021	7590	40.1	9750 (9420, 10100)	51.5 (49.7, 53.3)	-2160 (-2540, -1790)	0.78 (0.74, 0.81)
Serbia	2020	439	4.9	1040 (961, 1120)	11.6 (10.7, 12.5)	-600 (-689, -512)	0.42 (0.34, 0.50)
	2021	445	5	940 (855, 1020)	10.5 (9.6, 11.5)	-495 (-589, -401)	0.47 (0.38, 0.56)
Slovakia	2020	153	2.8	198 (165, 231)	3.6 (3, 4.2)	-44.9 (-85.6, -4.17)	0.77 (0.57, 0.97)
	2021	134	2.5	180 (145, 216)	3.3 (2.7, 4)	-46.4 (-88.5, -4.16)	0.74 (0.52, 0.97)
Slovenia	2020	75	3.6	89.7 (83.6, 95.8)	4.3 (4, 4.6)	-14.7 (-32.8, 3.34)	0.84 (0.62, 1.05)
	2021	79	3.8	83.6 (76.7, 90.4)	4 (3.7, 4.4)	-4.55 (-23.3, 14.2)	0.95 (0.72, 1.17)
Eastern Europe	2020	80300	38.6	99000 (95500, 102000)	47.6 (45.9, 49.2)	-18600 (-22100, -15200)	0.81 (0.78, 0.84)
	2021	79800	38.6	92400 (88600, 96200)	44.7 (42.9, 46.5)	-12600 (-16500, -8790)	0.86 (0.82, 0.90)
Belarus	2020	1510	16.1	1930 (1790, 2070)	20.6 (19.1, 22.1)	-419 (-579, -259)	0.78 (0.70, 0.86)
	2021	1480	15.9	1700 (1560, 1850)	18.3 (16.7, 19.9)	-220 (-386, -54.2)	0.87 (0.78, 0.96)
Estonia	2020	120	9.1	125 (117, 132)	9.5 (8.9, 10.1)	-4.57 (-27.4, 18.3)	0.96 (0.78, 1.15)
	2021	107	8.2	112 (104, 120)	8.5 (7.9, 9.2)	-4.96 (-26.9, 17)	0.96 (0.76, 1.16)
Latvia	2020			401 (354, 449)	21.2 (18.7, 23.7)		
	2021	255	13.6	359 (309, 410)	19.2 (16.5, 21.9)	-104 (-164, -45)	0.71 (0.55, 0.87)
Lithuania	2020	688	24.9	963 (894, 1030)	34.9 (32.4, 37.4)	-275 (-361, -189)	0.71 (0.63, 0.80)
	2021	628	23	893 (817, 970)	32.7 (30, 35.5)	-265 (-356, -175)	0.70 (0.61, 0.80)
Republic of Moldova	2020	1770	48.8	2610 (2480, 2740)	72.1 (68.6, 75.7)	-847 (-1000, -694)	0.68 (0.62, 0.73)
	2021	2070	57.5	2420 (2280, 2560)	67.4 (63.4, 71.4)	-354 (-523, -185)	0.85 (0.79, 0.92)
Russian Federation	2020	58700	40.3	69600 (66600, 72700)	47.8 (45.7, 49.9)	-10900 (-14000, -7810)	0.84 (0.80, 0.88)
	2021	56900	39.3	65000 (61600, 68400)	44.9 (42.5, 47.2)	-8080 (-11500, -4630)	0.88 (0.83, 0.93)
Ukraine	2020	17500	40.3	23300 (21800, 24800)	53.5 (50.1, 56.9)	-5750 (-7240, -4250)	0.75 (0.70, 0.81)
	2021	18300	42.5	21900 (20300, 23600)	50.9 (47, 54.7)	-3610 (-5290, -1930)	0.84 (0.76, 0.91)
High-income	2020	84600	7.8	98200 (96600, 99700)	9 (8.9, 9.2)	-13600 (-15200, -11900)	0.86 (0.85, 0.88)
	2021	85900	7.9	95100 (93200, 96900)	8.7 (8.5, 8.9)	-9140 (-11000, -7230)	0.90 (0.88, 0.92)
	2020	38500	20.7	43300 (42200, 44400)	23.3 (22.7, 23.9)	-4820 (-5980, -3650)	0.89 (0.86, 0.91)

High-income Asia Pacific	2021	35600	19.2	40700 (39500, 42000)	22 (21.3, 22.6)	-5090 (-6370, -3810)	0.87 (0.85, 0.9)
Brunei Darussalam	2020	314	70.3	261 (239, 283)	58.5 (53.6, 63.4)	52.8 (11.7, 93.9)	1.20 (1.05, 1.35)
	2021	235	52.1	270 (243, 298)	59.9 (53.8, 66)	-35.1 (-75.9, 5.59)	0.87 (0.72, 1.02)
Japan	2020	12700	9.9	13900 (13600, 14200)	10.8 (10.6, 11.1)	-1150 (-1540, -769)	0.92 (0.89, 0.94)
	2021	11500	9	13100 (12800, 13500)	10.3 (10, 10.5)	-1590 (-2000, -1180)	0.88 (0.85, 0.91)
Republic of Korea	2020	23100	44.7	26900 (25900, 27900)	52 (50.1, 54)	-3810 (-4880, -2740)	0.86 (0.82, 0.90)
	2021	21400	41.6	25100 (23900, 26200)	48.7 (46.4, 50.9)	-3660 (-4840, -2480)	0.85 (0.81, 0.90)
Singapore	2020	2360	41.4	2270 (2050, 2480)	39.8 (35.9, 43.6)	91.9 (-147, 331)	1.04 (0.93, 1.15)
	2021	2460	43	2270 (2000, 2530)	39.6 (35, 44.2)	193 (-89, 474)	1.08 (0.96, 1.21)
Australasia	2020	1930	6.3	1860 (1790, 1940)	6.1 (5.9, 6.4)	66.9 (-47.3, 181)	1.04 (0.97, 1.1)
	2021	1770	5.7	1910 (1820, 2000)	6.2 (5.9, 6.5)	-141 (-266, -16.8)	0.93 (0.86, 0.99)
Australia	2020	1610	6.4	1540 (1470, 1620)	6.1 (5.8, 6.4)	67.9 (-39.9, 176)	1.04 (0.97, 1.11)
	2021	1470	5.7	1590 (1500, 1680)	6.2 (5.8, 6.5)	-122 (-241, -3.2)	0.92 (0.85, 1.00)
New Zealand	2020	316	6.2	317 (304, 330)	6.2 (6, 6.5)	-1.09 (-38.3, 36.1)	1.00 (0.88, 1.11)
	2021	302	5.8	321 (305, 337)	6.2 (5.9, 6.5)	-19.4 (-57, 18.2)	0.94 (0.82, 1.06)
Western Europe	2020	22800	5.2	27000 (26100, 28000)	6.2 (6, 6.4)	-4190 (-5180, -3210)	0.84 (0.81, 0.88)
	2021	23500	5.4	26000 (24900, 27100)	5.9 (5.7, 6.2)	-2480 (-3650, -1320)	0.90 (0.86, 0.95)
Austria	2020	382	4.3	460 (400, 520)	5.1 (4.5, 5.8)	-78.1 (-149, -7.13)	0.83 (0.68, 0.98)
	2021	387	4.3	439 (371, 507)	4.9 (4.1, 5.6)	-52.1 (-131, 26.4)	0.88 (0.71, 1.05)
Belgium	2020	778	6.8	917 (858, 976)	8 (7.5, 8.5)	-139 (-219, -58.9)	0.85 (0.76, 0.94)
	2021	817	7.1	915 (845, 986)	8 (7.4, 8.6)	-98.2 (-188, -8.22)	0.89 (0.80, 0.99)
Denmark	2020	207	3.6	244 (220, 269)	4.2 (3.8, 4.6)	-37.4 (-74.7, -0.192)	0.85 (0.69, 1.00)
	2021	191	3.3	235 (207, 262)	4 (3.5, 4.5)	-43.5 (-82.4, -4.57)	0.81 (0.65, 0.98)
Finland	2020	174	3.1	214 (197, 231)	3.9 (3.6, 4.2)	-39.7 (-70.6, -8.85)	0.81 (0.66, 0.97)
	2021	170	3.1	207 (187, 227)	3.7 (3.4, 4.1)	-36.9 (-69.1, -4.74)	0.82 (0.66, 0.98)
France	2020	4400	6.6	4880 (4680, 5070)	7.4 (7.1, 7.7)	-481 (-715, -246)	0.90 (0.85, 0.95)
	2021	4040	6.1	4920 (4680, 5150)	7.4 (7, 7.8)	-876 (-1140, -608)	0.82 (0.77, 0.87)
Germany	2020	4010	4.7	4580 (4200, 4970)	5.4 (4.9, 5.8)	-572 (-975, -168)	0.88 (0.79, 0.96)
	2021	3790	4.4	4350 (3880, 4810)	5.1 (4.5, 5.6)	-556 (-1040, -76.2)	0.87 (0.77, 0.98)
Greece	2020	380	3.7	397 (361, 432)	3.9 (3.5, 4.2)	-17 (-69.1, 35.2)	0.96 (0.83, 1.09)
	2021	195	1.9	386 (345, 428)	3.8 (3.4, 4.2)	-191 (-241, -141)	0.50 (0.38, 0.63)
Ireland	2020	229	4.7	253 (222, 283)	5.1 (4.5, 5.8)	-23.8 (-66.3, 18.6)	0.91 (0.74, 1.07)
	2021	210	4.2	243 (208, 278)	4.9 (4.2, 5.6)	-32.7 (-77.7, 12.4)	0.87 (0.68, 1.05)
Israel	2020	158	1.7	217 (166, 268)	2.3 (1.8, 2.8)	-59 (-115, -2.54)	0.73 (0.49, 0.97)
	2021	213	2.2	204 (147, 261)	2.1 (1.5, 2.7)	8.9 (-55, 72.8)	1.04 (0.73, 1.36)
Italy	2020	2160	3.6	3200 (2670, 3730)	5.3 (4.4, 6.2)	-1040 (-1570, -502)	0.68 (0.54, 0.82)
	2021	2380	4	3040 (2370, 3710)	5.1 (4, 6.2)	-660 (-1340, 17.3)	0.78 (0.58, 0.98)
Netherlands	2020	614	3.6	758 (699, 817)	4.4 (4.1, 4.8)	-144 (-221, -67.9)	0.81 (0.71, 0.91)
	2021	671	3.9	745 (675, 814)	4.3 (3.9, 4.7)	-73.8 (-160, 12.4)	0.90 (0.79, 1.02)
Norway	2020	144	2.7	150 (129, 171)	2.8 (2.4, 3.2)	-5.91 (-37.4, 25.6)	0.96 (0.75, 1.17)

	2021	140	2.6	132 (110, 154)	2.4 (2, 2.8)	8.34 (-23.6, 40.3)	1.06 (0.82, 1.31)
Portugal	2020	1400	13.2	1590 (1440, 1730)	14.9 (13.6, 16.2)	-185 (-344, -24.7)	0.88 (0.79, 0.98)
	2021	1460	13.8	1500 (1340, 1670)	14.2 (12.7, 15.7)	-41.6 (-220, 136)	0.97 (0.86, 1.09)
Spain	2020	2950	6.4	4010 (3500, 4530)	8.8 (7.6, 9.9)	-1070 (-1590, -542)	0.73 (0.62, 0.85)
	2021	3370	7.4	3890 (3290, 4490)	8.5 (7.2, 9.9)	-521 (-1130, 86.3)	0.87 (0.72, 1.01)
Sweden	2020	316	3.1	463 (326, 601)	4.5 (3.2, 5.8)	-147 (-289, -5.38)	0.68 (0.42, 0.94)
	2021	344	3.3	435 (281, 590)	4.2 (2.7, 5.7)	-91.3 (-250, 67.7)	0.79 (0.46, 1.12)
Switzerland	2020	351	4	470 (362, 579)	5.3 (4.1, 6.5)	-119 (-234, -5.02)	0.75 (0.53, 0.96)
	2021	355	4	466 (337, 595)	5.2 (3.8, 6.7)	-111 (-245, 23.3)	0.76 (0.50, 1.02)
United Kingdom	2020	4180	6.2	4230 (3940, 4510)	6.3 (5.8, 6.7)	-42.3 (-354, 269)	0.99 (0.92, 1.06)
	2021	4800	7.1	3910 (3600, 4230)	5.8 (5.3, 6.2)	884 (541, 1230)	1.23 (1.13, 1.32)
Southern Latin America	2020	12700	18.9	15700 (15100, 16200)	23.2 (22.4, 24.1)	-2940 (-3550, -2340)	0.81 (0.78, 0.85)
	2021	15500	22.9	16200 (15500, 16900)	23.9 (22.8, 24.9)	-660 (-1400, 84.5)	0.96 (0.91, 1.00)
Argentina	2020	9320	20.6	11500 (11000, 12100)	25.4 (24.2, 26.6)	-2190 (-2760, -1620)	0.81 (0.76, 0.86)
	2021	11900	26.2	11900 (11200, 12600)	26.1 (24.6, 27.6)	39.2 (-669, 748)	1.00 (0.94, 1.06)
Chile	2020	2420	13	3090 (2950, 3230)	16.5 (15.8, 17.3)	-668 (-840, -496)	0.78 (0.73, 0.84)
	2021	2630	14	3200 (3020, 3380)	17 (16.1, 18)	-566 (-771, -361)	0.82 (0.76, 0.88)
Uruguay	2020	968	28.4	1050 (987, 1120)	30.9 (28.9, 32.8)	-84.7 (-174, 4.69)	0.92 (0.83, 1.00)
	2021	951	27.9	1080 (1000, 1170)	31.8 (29.4, 34.2)	-133 (-234, -31.6)	0.88 (0.79, 0.97)
High-income North America	2020	8610	2.3	10300 (10100, 10400)	2.8 (2.8, 2.8)	-1680 (-1910, -1450)	0.84 (0.81, 0.86)
	2021	9460	2.6	10200 (10000, 10400)	2.8 (2.7, 2.8)	-758 (-1020, -500)	0.93 (0.90, 0.95)
Canada	2020	1760	4.8	1940 (1850, 2020)	5.2 (5, 5.4)	-174 (-293, -55.7)	0.91 (0.85, 0.97)
	2021	1900	5.1	1990 (1890, 2100)	5.3 (5, 5.6)	-90.7 (-227, 45.4)	0.95 (0.89, 1.02)
United States of America	2020	6850	2.1	8350 (8240, 8470)	2.5 (2.5, 2.6)	-1500 (-1710, -1300)	0.82 (0.79, 0.84)
	2021	7560	2.3	8220 (8090, 8360)	2.5 (2.4, 2.5)	-668 (-887, -448)	0.92 (0.89, 0.95)
Latin America and Caribbean	2020	177000	30	217000 (212000, 222000)	36.8 (36, 37.6)	-40000 (-45000, -35000)	0.82 (0.80, 0.84)
	2021	191000	32.1	221000 (215000, 227000)	37.1 (36.1, 38.2)	-29900 (-36000, -23700)	0.86 (0.84, 0.89)
Caribbean	2020	15300	32.4	17800 (17100, 18600)	37.8 (36.2, 39.4)	-2550 (-3330, -1770)	0.86 (0.82, 0.90)
	2021	15100	31.8	17100 (16200, 18000)	36 (34.2, 37.8)	-2010 (-2890, -1120)	0.88 (0.83, 0.93)
Belize	2020	78	18.5	99.6 (62.7, 136)	23.7 (14.9, 32.4)	-21.6 (-62.3, 19.1)	0.78 (0.40, 1.17)
	2021	72	16.8	99.3 (55, 144)	23.1 (12.8, 33.4)	-27.3 (-74.5, 20)	0.73 (0.30, 1.15)
Cuba	2020	580	5.1	648 (608, 689)	5.7 (5.4, 6.1)	-68.2 (-130, -6.01)	0.89 (0.80, 0.99)
	2021	509	4.5	633 (585, 681)	5.6 (5.2, 6)	-124 (-189, -59.1)	0.80 (0.70, 0.91)
Dominican Republic	2020	2620	24	3540 (3320, 3760)	32.5 (30.5, 34.5)	-921 (-1160, -679)	0.74 (0.68, 0.80)
	2021	3370	30.6	3410 (3150, 3660)	30.9 (28.6, 33.2)	-32.4 (-311, 246)	0.99 (0.91, 1.07)
Guyana	2020	366	47.6	477 (427, 527)	62.1 (55.6, 68.6)	-111 (-174, -49)	0.77 (0.64, 0.89)
	2021	414	54.1	454 (397, 511)	59.4 (51.9, 66.8)	-40.1 (-110, 29.4)	0.91 (0.76, 1.06)
Haiti	2020	11300	89.2	12600 (11900, 13300)	100.1 (94.5, 105.6)	-1370 (-2100, -632)	0.89 (0.84, 0.95)
	2021	10400	80.9	12100 (11300, 12900)	93.8 (87.6, 100.1)	-1660 (-2490, -833)	0.86 (0.80, 0.93)
Jamaica	2020	61	2.2	87.7 (61.9, 114)	3.1 (2.2, 4.1)	-26.7 (-56.7, 3.31)	0.70 (0.37, 1.02)
	2021	57	2	85.9 (55.5, 116)	3.1 (2, 4.2)	-28.9 (-62.7, 4.93)	0.66 (0.31, 1.02)

Suriname	2020	103	17.8	142 (109, 176)	24.7 (18.8, 30.5)	-39.4 (-78.7, -0.149)	0.72 (0.46, 0.98)
	2021	88	15.2	143 (102, 184)	24.6 (17.6, 31.7)	-54.8 (-99.6, -9.95)	0.62 (0.34, 0.89)
Trinidad and Tobago	2020	220	15.8	213 (171, 255)	15.3 (12.3, 18.3)	7.05 (-43.7, 57.8)	1.03 (0.79, 1.27)
	2021	171	12.3	209 (160, 258)	15 (11.5, 18.5)	-38.1 (-93.5, 17.2)	0.82 (0.57, 1.07)
Andean Latin America	2020	35000	53.7	45500 (44500, 46500)	69.8 (68.3, 71.4)	-10500 (-11600, -9410)	0.77 (0.75, 0.79)
	2021	38300	57.9	45800 (44500, 47000)	69.2 (67.4, 71.1)	-7450 (-8740, -6170)	0.84 (0.81, 0.86)
Bolivia (Plurinational State of)	2020	6010	51.4	7240 (7070, 7400)	62 (60.5, 63.4)	-1230 (-1450, -1010)	0.83 (0.80, 0.86)
	2021	7070	59.9	7080 (6880, 7270)	60 (58.3, 61.6)	-5.56 (-261, 250)	1.00 (0.96, 1.04)
Ecuador	2020	5270	29.5	6420 (6010, 6820)	36 (33.7, 38.3)	-1150 (-1580, -715)	0.82 (0.76, 0.88)
	2021	5600	31	6620 (6110, 7130)	36.6 (33.8, 39.5)	-1020 (-1550, -493)	0.85 (0.77, 0.92)
Peru	2020	23800	66.6	31900 (31000, 32800)	89.3 (86.8, 91.8)	-8110 (-9060, -7150)	0.75 (0.72, 0.77)
	2021	25700	70.7	32100 (31000, 33200)	88.4 (85.4, 91.5)	-6420 (-7570, -5280)	0.80 (0.77, 0.83)
Central Latin America	2020	48800	19.4	64600 (62800, 66500)	25.7 (24.9, 26.4)	-15800 (-17700, -13900)	0.76 (0.73, 0.78)
	2021	55100	21.8	66800 (64400, 69300)	26.4 (25.4, 27.4)	-11700 (-14200, -9210)	0.82 (0.79, 0.86)
Colombia	2020	12100	24.8	14600 (14100, 15100)	29.9 (28.9, 31)	-2520 (-3060, -1970)	0.83 (0.79, 0.86)
	2021	13700	27.8	15100 (14400, 15700)	30.7 (29.4, 32)	-1400 (-2060, -730)	0.91 (0.86, 0.95)
Costa Rica	2020	342	7.2	380 (333, 427)	8 (7, 9)	-38 (-97.6, 21.6)	0.90 (0.74, 1.06)
	2021	350	7.4	372 (317, 428)	7.8 (6.7, 9)	-22.5 (-89.2, 44.2)	0.94 (0.76, 1.12)
El Salvador	2020	2040	31.8	3930 (3080, 4790)	61.2 (47.9, 74.5)	-1890 (-2750, -1030)	0.52 (0.36, 0.68)
	2021	1940	30	4260 (3130, 5390)	66 (48.5, 83.5)	-2320 (-3450, -1190)	0.45 (0.27, 0.64)
Guatemala	2020	2750	17.6	3690 (3490, 3900)	23.7 (22.3, 25)	-944 (-1180, -713)	0.74 (0.69, 0.80)
	2021	3090	19.6	3760 (3510, 4020)	23.9 (22.3, 25.5)	-675 (-952, -398)	0.82 (0.75, 0.89)
Honduras	2020	1850	18.6	2560 (2350, 2780)	25.8 (23.6, 27.9)	-715 (-945, -485)	0.72 (0.64, 0.80)
	2021	2060	20.4	2500 (2250, 2750)	24.7 (22.2, 27.2)	-435 (-702, -169)	0.83 (0.73, 0.93)
Mexico	2020	17600	13.7	24200 (24100, 24300)	18.8 (18.7, 18.9)	-6590 (-6870, -6310)	0.73 (0.71, 0.74)
	2021	21700	16.7	24600 (24500, 24700)	19 (19, 19.1)	-2970 (-3280, -2660)	0.88 (0.87, 0.89)
Nicaragua	2020	1890	28.6	2020 (1900, 2150)	30.7 (28.8, 32.6)	-139 (-291, 13.9)	0.93 (0.86, 1.01)
	2021	1840	27.5	1940 (1790, 2080)	29 (26.9, 31.2)	-101 (-269, 66.7)	0.95 (0.86, 1.03)
Panama	2020	1130	26.8	1640 (1230, 2060)	38.8 (29, 48.6)	-509 (-929, -88.5)	0.69 (0.47, 0.91)
	2021	1450	33.8	1640 (1140, 2140)	38.2 (26.6, 49.8)	-188 (-693, 317)	0.89 (0.59, 1.18)
Venezuela (Bolivarian Republic of)	2020	9150	33.9	11600 (10100, 13100)	43 (37.4, 48.5)	-2460 (-3970, -955)	0.79 (0.67, 0.90)
	2021	9090	34.1	12700 (10700, 14700)	47.7 (40.2, 55.2)	-3610 (-5610, -1600)	0.72 (0.58, 0.85)
Tropical Latin America	2020	78000	34.5	89100 (84800, 93500)	39.5 (37.6, 41.4)	-11200 (-15500, -6760)	0.87 (0.83, 0.92)
	2021	82200	36.1	90900 (85500, 96200)	39.9 (37.6, 42.3)	-8710 (-14100, -3330)	0.90 (0.85, 0.96)
Brazil	2020	75600	34.6	86300 (81900, 90600)	39.5 (37.5, 41.4)	-10700 (-15000, -6270)	0.88 (0.83, 0.92)
	2021	79400	36	87900 (82600, 93300)	39.9 (37.5, 42.3)	-8480 (-13900, -3100)	0.90 (0.85, 0.96)
Paraguay	2020	2360	33.3	2860 (2750, 2970)	40.4 (38.8, 41.9)	-499 (-644, -355)	0.83 (0.78, 0.88)
	2021	2730	38.1	2970 (2830, 3100)	41.4 (39.5, 43.3)	-231 (-402, -60.8)	0.92 (0.86, 0.98)
North Africa and Middle East	2020	158000	25.6	192000 (187000, 197000)	31.2 (30.4, 32)	-34100 (-39000, -29100)	0.82 (0.80, 0.85)
	2021	167000	26.9	197000 (191000, 204000)	31.7 (30.7, 32.7)	-29900 (-36200, -23600)	0.85 (0.82, 0.88)

Algeria	2020	17200	39.5	22600 (20500, 24800)	51.9 (47, 56.8)	-5400 (-7560, -3240)	0.76 (0.68, 0.85)
	2021	19000	43	22600 (20000, 25100)	51.1 (45.2, 56.9)	-3560 (-6150, -964)	0.84 (0.74, 0.95)
Bahrain	2020	193	12.7	147 (113, 181)	9.7 (7.5, 12)	46.1 (2.53, 89.7)	1.31 (1.00, 1.63)
	2021	196	12.8	140 (101, 180)	9.2 (6.6, 11.8)	55.5 (7.38, 104)	1.40 (1.02, 1.77)
Egypt	2020	6980	6.7	8270 (7760, 8770)	7.9 (7.5, 8.4)	-1280 (-1810, -756)	0.84 (0.78, 0.90)
	2021	6760	6.4	8330 (7720, 8940)	7.9 (7.3, 8.5)	-1570 (-2200, -939)	0.81 (0.74, 0.88)
Iran (Islamic Republic of)	2020	5990	7.1	8020 (7680, 8370)	9.4 (9, 9.8)	-2030 (-2410, -1650)	0.75 (0.70, 0.79)
	2021	6200	7.3	7640 (7240, 8030)	8.9 (8.5, 9.4)	-1440 (-1860, -1010)	0.81 (0.76, 0.86)
Iraq	2020	4990	12.3	6430 (6060, 6800)	15.8 (14.9, 16.8)	-1440 (-1840, -1050)	0.78 (0.72, 0.83)
	2021	5800	14.1	6140 (5720, 6570)	14.9 (13.9, 15.9)	-341 (-790, 107)	0.94 (0.87, 1.02)
Jordan	2020	250	2.1	473 (385, 560)	4 (3.2, 4.7)	-223 (-316, -130)	0.53 (0.37, 0.69)
	2021	219	1.8	483 (374, 591)	3.9 (3, 4.8)	-264 (-376, -152)	0.45 (0.28, 0.63)
Kuwait	2020	691	15.3	891 (742, 1040)	19.7 (16.4, 23)	-200 (-357, -42.7)	0.78 (0.62, 0.94)
	2021	736	15.8	908 (725, 1090)	19.5 (15.6, 23.5)	-172 (-363, 18.1)	0.81 (0.62, 1.00)
Lebanon	2020	658	11.9	645 (531, 759)	11.6 (9.6, 13.7)	12.8 (-112, 137)	1.02 (0.83, 1.21)
	2021	471	8.5	631 (496, 765)	11.4 (9, 13.8)	-160 (-301, -18.8)	0.75 (0.55, 0.95)
Libya	2020	1750	25.6	2150 (1470, 2830)	31.4 (21.5, 41.4)	-401 (-1090, 283)	0.81 (0.53, 1.10)
	2021	1940	28.3	2390 (1470, 3310)	34.7 (21.3, 48.1)	-443 (-1370, 482)	0.81 (0.46, 1.16)
Morocco	2020	29000	78.5	31500 (30500, 32500)	85.2 (82.5, 87.9)	-2460 (-3520, -1410)	0.92 (0.89, 0.95)
	2021	29300	78.9	31600 (30400, 32800)	85.1 (81.8, 88.4)	-2310 (-3570, -1050)	0.93 (0.89, 0.97)
Oman	2020	313	6.9	296 (220, 372)	6.5 (4.9, 8.2)	17.4 (-66.1, 101)	1.06 (0.77, 1.35)
	2021	231	4.9	288 (199, 377)	6.1 (4.2, 8)	-56.8 (-150, 37)	0.80 (0.50, 1.1)
Qatar	2020	861	29.7	931 (803, 1060)	32.1 (27.7, 36.5)	-69.6 (-210, 70.4)	0.93 (0.78, 1.07)
	2021	983	33	1050 (867, 1230)	35.2 (29.1, 41.2)	-63.8 (-254, 127)	0.94 (0.76, 1.12)
Saudi Arabia	2020	2450	6.6	2730 (2520, 2930)	7.4 (6.8, 7.9)	-278 (-503, -51.9)	0.90 (0.82, 0.98)
	2021	2580	6.8	2630 (2390, 2860)	7 (6.3, 7.6)	-51.4 (-307, 205)	0.98 (0.88, 1.08)
Syrian Arab Republic	2020	2640	18.9	2900 (2310, 3480)	20.7 (16.5, 24.9)	-257 (-853, 338)	0.91 (0.71, 1.11)
	2021	3290	23.4	3020 (2280, 3750)	21.5 (16.3, 26.7)	274 (-468, 1020)	1.09 (0.83, 1.35)
Tunisia	2020	2680	22.7	3310 (3050, 3580)	28.1 (25.9, 30.4)	-637 (-920, -353)	0.81 (0.73, 0.89)
	2021	2640	22.3	3320 (3000, 3650)	28.1 (25.4, 30.8)	-688 (-1020, -352)	0.79 (0.70, 0.89)
Türkiye	2020	8830	10.6	10800 (10600, 11000)	13 (12.8, 13.2)	-1970 (-2220, -1710)	0.82 (0.79, 0.84)
	2021	9050	10.8	10400 (10200, 10600)	12.4 (12.2, 12.7)	-1350 (-1630, -1070)	0.87 (0.84, 0.90)
United Arab Emirates	2020	62	0.6	76.9 (58.4, 95.4)	0.8 (0.6, 1)	-14.9 (-39, 9.19)	0.81 (0.50, 1.12)
	2021	66	0.7	78.4 (55.6, 101)	0.8 (0.6, 1.1)	-12.4 (-40.2, 15.4)	0.84 (0.50, 1.19)
Yemen	2020	8860	26.9	9750 (8100, 11400)	29.6 (24.6, 34.6)	-886 (-2540, 772)	0.91 (0.75, 1.07)
	2021	9370	27.8	9770 (7780, 11800)	29 (23.1, 35)	-405 (-2410, 1600)	0.96 (0.76, 1.16)
South Asia	2020	2160000	118.2	2950000 (2670000, 3220000)	161.3 (146.3, 176.3)	-787000 (-1060000, -513000)	0.73 (0.65, 0.81)
	2021	2640000	143	3130000 (2780000, 3490000)	169.7 (150.5, 189)	-493000 (-849000, -138000)	0.84 (0.74, 0.95)
Afghanistan	2020	45800	151.6	59400 (55800, 62900)	196.4 (184.6, 208.2)	-13500 (-17100, -9960)	0.77 (0.72, 0.83)
	2021	50300	161.2	65500 (60700, 70300)	209.9 (194.5, 225.2)	-15200 (-20000, -10400)	0.77 (0.70, 0.83)
Bangladesh	2020	230000	140.8	311000 (301000, 320000)	190 (184, 196)	-80500 (-90400, -70700)	0.74 (0.71, 0.77)

	2021	307000	186.3	335000 (323000, 348000)	203.7 (195.9, 211.6)	-28700 (-41700, -15800)	0.91 (0.88, 0.95)
Bhutan	2020	911	120.1	900 (755, 1050)	118.7 (99.5, 137.9)	10.7 (-147, 168)	1.01 (0.84, 1.19)
	2021	854	112.8	873 (704, 1040)	115.4 (93, 137.8)	-19.5 (-199, 160)	0.98 (0.77, 1.18)
India	2020	1630000	116.2	2240000 (1970000, 2510000)	159.5 (140.2, 178.9)	-608000 (-879000, -336000)	0.73 (0.62, 0.83)
	2021	1970000	139	2390000 (2040000, 2740000)	168.9 (144.1, 193.8)	-424000 (-776000, -72400)	0.82 (0.69, 0.96)
Nepal	2020	27300	88.5	30300 (28700, 32000)	98.4 (93.1, 103.7)	-3060 (-4720, -1400)	0.90 (0.85, 0.95)
	2021	28300	90.8	29800 (27900, 31700)	95.6 (89.5, 101.8)	-1520 (-3460, 428)	0.95 (0.89, 1.01)
Pakistan	2020	273000	118.4	369000 (331000, 407000)	160.1 (143.6, 176.6)	-96200 (-134000, -58100)	0.74 (0.65, 0.83)
	2021	339000	144	378000 (331000, 425000)	160.5 (140.5, 180.5)	-38800 (-85900, 8290)	0.90 (0.78, 1.02)
Sub-Saharan Africa	2020	1370000	123.9	1410000 (1380000, 1450000)	127.8 (124.8, 130.8)	-43900 (-77200, -10600)	0.97 (0.95, 0.99)
	2021	1480000	130.6	1450000 (1410000, 1500000)	128.3 (124.6, 131.9)	26100 (-15500, 67700)	1.02 (0.99, 1.05)
Central Sub-Saharan Africa	2020	294000	220.3	295000 (278000, 312000)	220.6 (208, 233.2)	-383 (-17300, 16500)	1.00 (0.94, 1.06)
	2021	308000	225.1	315000 (293000, 336000)	229.8 (213.8, 245.7)	-6380 (-28300, 15500)	0.98 (0.91, 1.05)
Angola	2020	63100	199.3	70600 (60600, 80500)	222.7 (191.2, 254.2)	-7410 (-17400, 2580)	0.89 (0.76, 1.03)
	2021	61700	188.6	73000 (60500, 85500)	223.3 (185.1, 261.5)	-11300 (-23800, 1170)	0.84 (0.69, 1.00)
Central African Republic	2020	12600	233.8	12200 (10800, 13500)	225.9 (200.9, 250.9)	427 (-939, 1790)	1.04 (0.92, 1.15)
	2021	13200	241	12700 (11000, 14400)	232.1 (201, 263.2)	487 (-1230, 2200)	1.04 (0.90, 1.18)
Congo	2020	11200	210.8	11100 (10100, 12200)	210 (190.1, 230)	40.7 (-1040, 1120)	1.00 (0.91, 1.10)
	2021	12000	222.2	11300 (9970, 12500)	208.7 (184.9, 232.6)	724 (-581, 2030)	1.06 (0.94, 1.18)
Democratic Republic of the Congo	2020	201000	228.5	194000 (180000, 207000)	220.3 (205, 235.6)	7210 (-6270, 20700)	1.04 (0.97, 1.11)
	2021	214000	238.2	211000 (193000, 228000)	233.9 (214.1, 253.6)	3870 (-13900, 21700)	1.02 (0.93, 1.10)
Equatorial Guinea	2020	1510	102.6	1540 (1370, 1710)	104.5 (93, 116)	-27.4 (-213, 158)	0.98 (0.86, 1.10)
	2021	1880	124.2	1590 (1370, 1800)	105 (90.6, 119.3)	290 (57.2, 524)	1.18 (1.03, 1.34)
Gabon	2020	4880	272.4	5500 (5100, 5900)	307.1 (284.8, 329.4)	-622 (-1040, -200)	0.89 (0.81, 0.96)
	2021	5070	279.3	5490 (5010, 5970)	302.3 (275.9, 328.8)	-418 (-918, 82.5)	0.92 (0.84, 1.01)
Eastern Sub-Saharan Africa	2020	562000	134.8	603000 (582000, 623000)	144.6 (139.6, 149.6)	-41000 (-61900, -20100)	0.93 (0.90, 0.97)
	2021	595000	139.6	631000 (604000, 658000)	148.2 (141.8, 154.5)	-36400 (-63300, -9410)	0.94 (0.90, 0.98)
Burundi	2020	7100	55.5	7200 (6490, 7900)	56.2 (50.7, 61.7)	-93.8 (-818, 630)	0.99 (0.89, 1.09)
	2021	6790	51.3	7180 (6330, 8020)	54.3 (47.9, 60.7)	-390 (-1250, 470)	0.95 (0.83, 1.06)
Comoros	2020	156	21.2	166 (115, 217)	22.6 (15.7, 29.4)	-9.98 (-66.3, 46.3)	0.94 (0.61, 1.27)
	2021	134	18	172 (108, 235)	23.1 (14.5, 31.6)	-37.7 (-105, 29.7)	0.78 (0.42, 1.14)
Djibouti	2020	1700	138.5	1680 (1440, 1920)	136.7 (117.1, 156.3)	22.4 (-231, 276)	1.01 (0.86, 1.17)
	2021	1800	142.9	1540 (1270, 1800)	122.1 (101.2, 143.1)	262 (-14.4, 537)	1.17 (0.98, 1.36)
Eritrea	2020	1590	24.7	1660 (1460, 1860)	25.7 (22.6, 28.8)	-65.3 (-280, 149)	0.96 (0.83, 1.09)
	2021	1600	24.3	1560 (1330, 1780)	23.6 (20.2, 27)	45.5 (-192, 283)	1.03 (0.88, 1.18)
Ethiopia	2020	108000	101.4	109000 (99100, 119000)	102.1 (92.9, 111.4)	-801 (-10700, 9090)	0.99 (0.90, 1.08)
	2021	105000	96	106000 (94300, 117000)	97.1 (86.6, 107.7)	-1230 (-12800, 10300)	0.99 (0.88, 1.10)
Kenya	2020	71100	144	84900 (74000, 95700)	171.9 (149.9, 193.9)	-13800 (-24700, -2920)	0.84 (0.72, 0.95)
	2021	76000	151.8	84500 (71400, 97500)	168.7 (142.7, 194.7)	-8440 (-21500, 4590)	0.90 (0.75, 1.05)
Madagascar	2020	36100	129.2	37600 (35500, 39700)	134.5 (127, 142)	-1460 (-3580, 671)	0.96 (0.91, 1.02)
	2021	40000	140.2	39400 (36700, 42100)	137.9 (128.6, 147.3)	637 (-2050, 3330)	1.02 (0.95, 1.08)

Malawi	2020	15100	79.6	15800 (14400, 17200)	83.2 (75.8, 90.5)	-686 (-2100, 733)	0.96 (0.87, 1.04)
	2021	14400	74	15600 (14000, 17300)	80.4 (71.9, 89)	-1250 (-2930, 427)	0.92 (0.82, 1.02)
Mauritius	2020	101	7.9	120 (112, 128)	9.4 (8.8, 10.1)	-19 (-40.3, 2.43)	0.84 (0.65, 1.03)
	2021	91	7.2	119 (109, 129)	9.3 (8.6, 10.1)	-27.7 (-48.8, -6.51)	0.77 (0.57, 0.96)
Mozambique	2020	96200	317.6	111000 (102000, 121000)	367.7 (335.7, 399.7)	-15200 (-24900, -5460)	0.86 (0.78, 0.94)
	2021	97900	315.1	124000 (111000, 137000)	399.2 (356.8, 441.7)	-26100 (-39300, -12900)	0.79 (0.69, 0.88)
Rwanda	2020	5510	42.4	5770 (5580, 5960)	44.4 (42.9, 45.8)	-259 (-499, -19.5)	0.96 (0.91, 1.00)
	2021	5220	39.3	5780 (5550, 6010)	43.6 (41.8, 45.3)	-563 (-833, -293)	0.90 (0.86, 0.95)
Somalia	2020	17200	82.7	18200 (17100, 19300)	87.6 (82.3, 93)	-1030 (-2170, 114)	0.94 (0.88, 1.00)
	2021	17400	80.6	19200 (17800, 20600)	88.8 (82.3, 95.4)	-1770 (-3210, -327)	0.91 (0.84, 0.98)
United Republic of Tanzania	2020	84800	148.3	83900 (78800, 89000)	146.7 (137.8, 155.6)	903 (-4220, 6030)	1.01 (0.95, 1.07)
	2021	86700	148.3	88800 (82100, 95500)	152 (140.5, 163.4)	-2120 (-8850, 4620)	0.98 (0.90, 1.05)
Uganda	2020	60900	143.9	71400 (62400, 80400)	168.8 (147.6, 190)	-10500 (-19500, -1530)	0.85 (0.74, 0.97)
	2021	74800	172.7	80300 (67300, 93400)	185.5 (155.3, 215.7)	-5540 (-18600, 7540)	0.93 (0.77, 1.09)
Zambia	2020	40000	210.2	34300 (32400, 36100)	180.1 (170.5, 189.7)	5740 (3870, 7610)	1.17 (1.11, 1.23)
	2021	50100	256.6	33400 (31300, 35500)	171.1 (160.2, 182.1)	16700 (14500, 18900)	1.50 (1.42, 1.58)
Southern Sub-Saharan Africa	2020	223000	279.3	235000 (219000, 250000)	294.6 (275.3, 314)	-12200 (-27700, 3210)	0.95 (0.88, 1.01)
	2021	204000	254	218000 (201000, 235000)	271.7 (250.4, 293)	-14200 (-31400, 2910)	0.93 (0.86, 1.01)
Botswana	2020	2480	105.1	3480 (2720, 4240)	147.2 (115.2, 179.2)	-995 (-1760, -233)	0.71 (0.53, 0.90)
	2021	2350	98.2	3150 (2340, 3970)	131.7 (97.6, 165.9)	-803 (-1630, 20.1)	0.75 (0.52, 0.97)
Lesotho	2020	4560	243.6	6300 (5670, 6930)	336.2 (302.7, 369.7)	-1730 (-2380, -1090)	0.72 (0.64, 0.81)
	2021	4490	239.5	5970 (5250, 6680)	318.3 (280.3, 356.3)	-1480 (-2200, -751)	0.75 (0.65, 0.86)
Namibia	2020	6510	270.6	7530 (7060, 8010)	313.2 (293.3, 333)	-1030 (-1530, -522)	0.86 (0.80, 0.93)
	2021	6700	275.7	7240 (6690, 7790)	298 (275.3, 320.6)	-541 (-1110, 32.1)	0.93 (0.85, 1.00)
South Africa	2020	191000	338.2	194000 (179000, 210000)	343.9 (316.9, 371)	-3270 (-18600, 12000)	0.98 (0.91, 1.06)
	2021	172000	302.9	180000 (163000, 197000)	317.2 (287.4, 347.1)	-8170 (-25100, 8810)	0.95 (0.86, 1.05)
Eswatini	2020	2140	186.4	2190 (1840, 2530)	190.1 (159.8, 220.4)	-42.6 (-403, 318)	0.98 (0.82, 1.14)
	2021	1930	167.4	1870 (1520, 2230)	162.3 (131.5, 193)	59.1 (-306, 425)	1.03 (0.83, 1.23)
Zimbabwe	2020	15700	102.3	20900 (19200, 22600)	135.9 (124.8, 146.9)	-5160 (-6880, -3450)	0.75 (0.68, 0.82)
	2021	16300	104.6	19600 (17700, 21500)	125.7 (113.5, 137.9)	-3300 (-5220, -1370)	0.83 (0.74, 0.92)
Western Sub-Saharan Africa	2020	292000	61.3	282000 (270000, 294000)	59.2 (56.7, 61.8)	9730 (-2460, 21900)	1.03 (0.99, 1.08)
	2021	372000	76	289000 (274000, 305000)	59.1 (56, 62.2)	83100 (67700, 98400)	1.29 (1.23, 1.35)
Benin	2020	3910	29.9	4060 (3700, 4420)	31 (28.3, 33.8)	-155 (-536, 227)	0.96 (0.87, 1.05)
	2021	3710	27.5	4090 (3650, 4530)	30.3 (27.1, 33.6)	-382 (-837, 73.3)	0.91 (0.80, 1.01)
Burkina Faso	2020	5690	25.7	5480 (4880, 6080)	24.7 (22, 27.4)	212 (-406, 830)	1.04 (0.92, 1.15)
	2021	6880	30.2	5460 (4740, 6190)	24 (20.8, 27.2)	1420 (678, 2160)	1.26 (1.11, 1.41)
Cameroon	2020	22100	71.6	23500 (22500, 24400)	76 (72.7, 79.2)	-1360 (-2390, -325)	0.94 (0.90, 0.99)
	2021	22400	70.5	23000 (21800, 24200)	72.4 (68.7, 76)	-587 (-1790, 616)	0.97 (0.92, 1.03)
Cabo Verde	2020	207	37.2	188 (172, 204)	33.9 (31, 36.7)	18.7 (-13.7, 51.1)	1.10 (0.93, 1.27)
	2021	182	32.5	176 (158, 194)	31.4 (28.2, 34.6)	6.29 (-25.6, 38.2)	1.04 (0.86, 1.22)
Chad	2020	12800	74.9	13400 (12100, 14800)	78.5 (70.7, 86.4)	-629 (-1990, 735)	0.95 (0.85, 1.05)

	2021	13600	76.9	13800 (12200, 15500)	77.9 (68.5, 87.3)	-183 (-1870, 1510)	0.99 (0.87, 1.11)
Côte d'Ivoire	2020	19700	72.4	19900 (18800, 20900)	72.9 (69.2, 76.6)	-138 (-1190, 916)	0.99 (0.94, 1.05)
	2021	20700	74.4	19300 (18100, 20500)	69.3 (65, 73.6)	1420 (200, 2640)	1.07 (1.01, 1.14)
Gambia	2020	2400	102.8	2550 (2390, 2720)	109.2 (102.1, 116.3)	-149 (-341, 43.2)	0.94 (0.87, 1.02)
	2021	2250	94.1	2570 (2360, 2770)	107.2 (98.8, 115.6)	-312 (-534, -89.8)	0.88 (0.80, 0.96)
Ghana	2020	12700	37.9	14000 (13300, 14600)	41.7 (39.9, 43.5)	-1280 (-1930, -636)	0.91 (0.86, 0.95)
	2021	13400	39.1	13800 (13100, 14500)	40.3 (38.2, 42.4)	-402 (-1160, 356)	0.97 (0.92, 1.03)
Guinea	2020	15600	119.3	16600 (15600, 17500)	126.7 (119.5, 134)	-970 (-1950, 6.73)	0.94 (0.88, 1.00)
	2021	18800	139.6	17600 (16400, 18800)	130.9 (121.8, 139.9)	1180 (-67.7, 2420)	1.07 (0.99, 1.14)
Guinea-Bissau	2020	2540	126	2270 (2050, 2490)	112.6 (101.6, 123.6)	270 (27.2, 513)	1.12 (1.01, 1.23)
	2021	2490	120.6	2290 (2020, 2560)	110.8 (97.7, 123.8)	203 (-83.3, 490)	1.09 (0.96, 1.22)
Liberia	2020	6960	130.6	8530 (6530, 10500)	160.1 (122.5, 197.7)	-1570 (-3580, 436)	0.82 (0.60, 1.03)
	2021	7330	134.2	8970 (6420, 11500)	164.2 (117.6, 210.9)	-1640 (-4190, 914)	0.82 (0.56, 1.07)
Mali	2020	6920	29.8	7230 (6660, 7810)	31.1 (28.6, 33.6)	-312 (-909, 284)	0.96 (0.88, 1.04)
	2021	7280	30.2	7430 (6720, 8140)	30.8 (27.9, 33.8)	-146 (-877, 586)	0.98 (0.88, 1.08)
Mauritania	2020	2340	54.4	2510 (2330, 2680)	58.2 (54.2, 62.3)	-164 (-361, 32.9)	0.93 (0.86, 1.01)
	2021	2440	55.5	2540 (2330, 2750)	57.8 (53, 62.6)	-102 (-334, 131)	0.96 (0.87, 1.05)
Niger	2020	11400	47.2	10800 (9780, 11700)	44.6 (40.6, 48.7)	620 (-376, 1620)	1.06 (0.96, 1.15)
	2021	12800	51.1	10800 (9590, 11900)	43 (38.3, 47.7)	2020 (824, 3220)	1.19 (1.07, 1.31)
Nigeria	2020	136000	60.3	116000 (105000, 128000)	51.6 (46.5, 56.7)	19600 (8070, 31200)	1.17 (1.06, 1.28)
	2021	205000	88.6	121000 (106000, 135000)	52.3 (46, 58.6)	83800 (69200, 98400)	1.69 (1.54, 1.85)
Sao Tome and Principe	2020	92	43.1	145 (114, 176)	68.1 (53.6, 82.5)	-53.3 (-89.4, -17.1)	0.63 (0.40, 0.87)
	2021	82	37.9	142 (106, 179)	65.6 (48.9, 82.4)	-60.2 (-101, -19.8)	0.58 (0.32, 0.83)
Senegal	2020	12800	82.3	13200 (12900, 13500)	85 (83.1, 86.9)	-428 (-797, -59)	0.97 (0.94, 1.00)
	2021	13400	84.3	13200 (12900, 13600)	83.3 (81.1, 85.6)	153 (-267, 574)	1.01 (0.98, 1.04)
Sierra Leone	2020	15700	182.1	19300 (17600, 21000)	224.3 (204.6, 243.9)	-3640 (-5350, -1920)	0.81 (0.73, 0.89)
	2021	17600	197.9	20800 (18600, 23000)	234.7 (209.7, 259.6)	-3260 (-5490, -1030)	0.84 (0.74, 0.94)
Togo	2020	2330	28.4	2510 (2340, 2690)	30.7 (28.5, 32.8)	-186 (-386, 13.8)	0.93 (0.85, 1.00)
	2021	2440	29.1	2490 (2280, 2700)	29.8 (27.3, 32.3)	-54.1 (-285, 177)	0.98 (0.89, 1.07)
South Sudan	2020	16000	169.7	19700 (17200, 22300)	209.7 (182.2, 237.1)	-3770 (-6360, -1170)	0.81 (0.69, 0.93)
	2021	17500	180.6	24000 (20100, 27800)	247.9 (208.1, 287.8)	-6510 (-10400, -2650)	0.73 (0.59, 0.87)
Sudan	2020	17400	40.7	20400 (19400, 21400)	47.8 (45.4, 50.2)	-3040 (-4100, -1990)	0.85 (0.80, 0.90)
	2021	18300	42.1	20400 (19200, 21700)	47.1 (44.2, 49.9)	-2190 (-3450, -922)	0.89 (0.83, 0.95)

Table S2. Difference in observed to expected tuberculosis diagnoses during the COVID-19 pandemic for 65 ≥ years age group and under 65 age group in 2020 and 2021 for 155 countries.

		65 ≥ years		65 < years		
**Location**	**Year**	**Difference**	**OE ratio**	**Difference**	**OE ratio**	**Relative Risk**
Global	2020	-272000 (-316000, -229000)	0.71 (0.67, 0.75)	-1260000 (-1640000, -870000)	0.81 (0.75, 0.86)	0.88 (0.81, 0.96)
	2021	-271000 (-336000, -207000)	0.73 (0.68, 0.79)	-1160000 (-1680000, -630000)	0.83 (0.76, 0.90)	0.88 (0.79, 0.98)
Southeast Asia, East Asia, and Oceania	2020	-128000 (-165000, -90900)	0.70 (0.63, 0.77)	-509000 (-677000, -341000)	0.75 (0.68, 0.82)	0.94 (0.82, 1.07)
	2021	-174000 (-231000, -117000)	0.63 (0.53, 0.73)	-705000 (-949000, -462000)	0.68 (0.59, 0.77)	0.93 (0.76, 1.14)
East Asia	2020	-35400 (-46500, -24300)	0.82 (0.77, 0.87)	-58900 (-85000, -32800)	0.91 (0.87, 0.95)	0.90 (0.84, 0.97)
	2021	-34000 (-47500, -20400)	0.83 (0.77, 0.89)	-138000 (-169000, -108000)	0.79 (0.74, 0.83)	1.06 (0.97, 1.15)
China	2020	-35000 (-46100, -24000)	0.82 (0.77, 0.87)	-52000 (-76400, -27600)	0.91 (0.87, 0.95)	0.90 (0.84, 0.98)
	2021	-33700 (-47200, -20100)	0.83 (0.77, 0.89)	-129000 (-158000, -101000)	0.77 (0.72, 0.81)	1.08 (0.98, 1.19)
Democratic People's Republic of Korea	2020	-390 (-1260, 478)	0.91 (0.71, 1.10)	-6930 (-16200, 2340)	0.93 (0.83, 1.02)	0.98 (0.78, 1.25)
	2021	-313 (-1340, 718)	0.92 (0.69, 1.16)	-9080 (-20200, 2080)	0.90 (0.79, 1.02)	1.03 (0.77, 1.34)
Southeast Asia	2020	-92400 (-128000, -57200)	0.60 (0.48, 0.72)	-450000 (-616000, -284000)	0.67 (0.58, 0.77)	0.89 (0.70, 1.14)
	2021	-140000 (-196000, -84900)	0.48 (0.34, 0.62)	-567000 (-809000, -325000)	0.63 (0.51, 0.76)	0.76 (0.54, 1.12)
Cambodia	2020	2.31 (-606, 611)	1.00 (0.89, 1.11)	2610 (-544, 5770)	1.13 (0.96, 1.29)	0.89 (0.74, 1.06)
	2021	-1710 (-2440, -977)	0.69 (0.58, 0.80)	-1320 (-4880, 2240)	0.93 (0.75, 1.11)	0.74 (0.57, 0.95)
Indonesia	2020	-34700 (-44600, -24800)	0.50 (0.40, 0.60)	-176000 (-263000, -89500)	0.69 (0.56, 0.81)	0.73 (0.56, 0.97)
	2021	-47900 (-62400, -33500)	0.42 (0.31, 0.54)	-184000 (-301000, -67000)	0.71 (0.55, 0.86)	0.60 (0.43, 0.85)
Lao People's Democratic Republic	2020	68.6 (-88.1, 225)	1.04 (0.95, 1.13)	249 (-73.9, 572)	1.04 (0.99, 1.10)	1.00 (0.90, 1.10)
	2021	-822 (-1040, -602)	0.60 (0.52, 0.68)	-1520 (-1950, -1090)	0.76 (0.71, 0.82)	0.79 (0.68, 0.93)
Malaysia	2020	-522 (-676, -369)	0.87 (0.83, 0.90)	-1850 (-3230, -465)	0.92 (0.87, 0.98)	0.94 (0.87, 1.01)
	2021	-1030 (-1220, -837)	0.74 (0.70, 0.78)	-4000 (-5710, -2290)	0.83 (0.77, 0.90)	0.89 (0.81, 0.97)
Maldives	2020	-1.16 (-3.39, 1.07)	0.89 (0.68, 1.09)	-54.1 (-85.3, -22.9)	0.64 (0.48, 0.81)	1.37 (0.97, 1.94)
	2021	-0.547 (-2.83, 1.73)	0.94 (0.68, 1.19)	-80.1 (-120, -40.6)	0.50 (0.32, 0.67)	1.88 (1.21, 2.92)
Myanmar	2020	-4820 (-5920, -3720)	0.73 (0.68, 0.78)	-25100 (-33200, -16900)	0.80 (0.74, 0.85)	0.92 (0.82, 1.03)
	2021	-10800 (-12300, -9400)	0.43 (0.38, 0.48)	-65700 (-75800, -55700)	0.46 (0.41, 0.52)	0.92 (0.78, 1.12)
Philippines	2020	-50100 (-83700, -16500)	0.43 (0.18, 0.68)	-239000 (-380000, -97800)	0.50 (0.29, 0.71)	0.87 (0.43, 1.79)
	2021	-66400 (-120000, -13200)	0.40 (0.10, 0.71)	-267000 (-478000, -55700)	0.53 (0.26, 0.80)	0.75 (0.29, 1.88)
Sri Lanka	2020	-345 (-446, -244)	0.81 (0.76, 0.86)	-780 (-1120, -439)	0.88 (0.83, 0.93)	0.92 (0.85, 1.00)
	2021	-580 (-705, -454)	0.69 (0.63, 0.75)	-889 (-1290, -492)	0.86 (0.80, 0.92)	0.81 (0.72, 0.90)
Thailand	2020	-1420 (-3220, 380)	0.94 (0.86, 1.01)	-8340 (-11000, -5690)	0.89 (0.86, 0.92)	1.05 (0.96, 1.16)
	2021	-5490 (-8110, -2860)	0.77 (0.67, 0.87)	-27400 (-31300, -23500)	0.67 (0.63, 0.71)	1.14 (0.99, 1.31)
Timor-Leste	2020	-227 (-242, -211)	0.67 (0.65, 0.69)	-720 (-1120, -320)	0.79 (0.69, 0.90)	0.84 (0.74, 0.96)
	2021	-196 (-221, -172)	0.75 (0.72, 0.77)	-984 (-1540, -429)	0.73 (0.59, 0.86)	1.03 (0.85, 1.23)
Viet Nam	2020	-259 (-2600, 2080)	0.99 (0.87, 1.11)	-1170 (-5220, 2870)	0.99 (0.94, 1.03)	1.00 (0.88, 1.15)
	2021	-5280 (-8540, -2020)	0.74 (0.59, 0.88)	-13800 (-19100, -8400)	0.83 (0.77, 0.89)	0.89 (0.72, 1.08)
Oceania	2020	-37.4 (-72.6, -2.26)	0.74 (0.53, 0.95)	-138 (-330, 53.8)	0.91 (0.79, 1.03)	0.81 (0.58, 1.10)
	2021	-61.9 (-111, -12.6)	0.60 (0.35, 0.85)	-359 (-604, -113)	0.77 (0.63, 0.91)	0.77 (0.50, 1.25)
Fiji	2020	-14.2 (-31, 2.66)	0.73 (0.45, 1.00)	5.55 (-78.4, 89.5)	1.01 (0.81, 1.22)	0.72 (0.48, 1.12)

	2021	-29 (-51, -7.07)	0.48 (0.21, 0.75)	-131 (-239, -22.2)	0.70 (0.50, 0.91)	0.68 (0.36, 1.27)
Kiribati	2020	4 (-5.95, 13.9)	1.13 (0.78, 1.49)	-2.2 (-109, 105)	0.99 (0.69, 1.30)	1.14 (0.72, 1.76)
	2021	-10.8 (-23.5, 1.96)	0.66 (0.33, 0.99)	-33.4 (-158, 91.2)	0.90 (0.56, 1.25)	0.73 (0.39, 1.31)
Marshall Islands	2020	-13.2 (-39.5, 13)	0.40 (0.00, 1.15)	-109 (-233, 15.9)	0.57 (0.20, 0.94)	0.71 (0.10, 5.13)
	2021	-21.4 (-60.8, 18.1)	0.22 (0.00, 0.89)	-174 (-346, -2.96)	0.39 (0.02, 0.77)	0.56 (0.02, 12.22)
Micronesia (Federated States of)	2020	-3.56 (-7.5, 0.376)	0.46 (0.05, 0.86)	16.4 (-18.5, 51.4)	1.21 (0.72, 1.69)	0.38 (0.14, 1.06)
	2021					
Solomon Islands	2020	-0.43 (-11.3, 10.4)	0.98 (0.39, 1.56)	-58.7 (-97.6, -19.7)	0.84 (0.74, 0.94)	1.17 (0.63, 2.10)
	2021	14.3 (1.78, 26.8)	1.81 (0.86, 2.76)	-25.3 (-72.4, 21.9)	0.93 (0.81, 1.06)	1.94 (1.11, 3.23)
Vanuatu	2020	-10 (-15.8, -4.23)	0.29 (0.06, 0.51)	9.6 (6, 13.2)	1.14 (1.08, 1.19)	0.25 (0.11, 0.56)
	2021	-8.72 (-16.1, -1.34)	0.41 (0.09, 0.73)	-1.99 (-6.02, 2.04)	0.97 (0.91, 1.03)	0.42 (0.20, 0.92)
Central Europe, Eastern Europe, and Central Asia	2020	-5350 (-6240, -4460)	0.72 (0.68, 0.76)	-33600 (-42300, -24900)	0.78 (0.73, 0.83)	0.92 (0.85, 1.00)
	2021	-4680 (-5760, -3610)	0.75 (0.70, 0.80)	-25100 (-34900, -15200)	0.83 (0.77, 0.89)	0.91 (0.82, 1.01)
Central Asia	2020	-1760 (-2060, -1450)	0.72 (0.68, 0.76)	-8080 (-10700, -5420)	0.81 (0.76, 0.87)	0.89 (0.81, 0.98)
	2021	-1660 (-2050, -1260)	0.75 (0.70, 0.80)	-5430 (-8440, -2410)	0.87 (0.80, 0.94)	0.87 (0.78, 0.96)
Armenia	2020	-6.11 (-16.2, 3.94)	0.90 (0.74, 1.06)	-155 (-198, -113)	0.70 (0.63, 0.77)	1.29 (1.05, 1.59)
	2021	-6.81 (-17.3, 3.71)	0.87 (0.69, 1.06)	-81 (-125, -37.1)	0.82 (0.73, 0.91)	1.07 (0.84, 1.35)
Azerbaijan	2020	-97.7 (-134, -61.7)	0.65 (0.55, 0.76)	-774 (-939, -609)	0.75 (0.71, 0.80)	0.87 (0.72, 1.02)
	2021	-113 (-160, -66)	0.63 (0.51, 0.75)	-651 (-839, -464)	0.78 (0.73, 0.84)	0.80 (0.65, 0.98)
Georgia	2020	-73.5 (-120, -26.9)	0.74 (0.59, 0.88)	-253 (-373, -133)	0.86 (0.80, 0.92)	0.85 (0.70, 1.04)
	2021	-96 (-150, -41.6)	0.64 (0.48, 0.81)	-291 (-424, -158)	0.83 (0.76, 0.90)	0.77 (0.59, 1.00)
Kazakhstan	2020	-298 (-500, -96.7)	0.79 (0.67, 0.92)	-720 (-2660, 1220)	0.93 (0.74, 1.12)	0.86 (0.67, 1.11)
	2021	-228 (-480, 24.7)	0.85 (0.69, 1.00)	-15.8 (-2180, 2150)	1.00 (0.76, 1.23)	0.85 (0.61, 1.16)
Kyrgyzstan	2020	-438 (-597, -280)	0.57 (0.45, 0.69)	-2020 (-2830, -1210)	0.64 (0.53, 0.76)	0.88 (0.68, 1.15)
	2021	-537 (-762, -311)	0.55 (0.40, 0.69)	-1670 (-2640, -709)	0.70 (0.56, 0.85)	0.78 (0.56, 1.06)
Mongolia	2020	-32 (-57.9, -6.14)	0.87 (0.77, 0.97)	-173 (-514, 168)	0.95 (0.87, 1.04)	0.91 (0.77, 1.05)
	2021	-103 (-136, -70.6)	0.59 (0.49, 0.69)	-1190 (-1590, -784)	0.68 (0.60, 0.77)	0.86 (0.69, 1.06)
Tajikistan	2020	-180 (-262, -98.3)	0.69 (0.58, 0.81)	-1600 (-1920, -1290)	0.70 (0.65, 0.75)	0.99 (0.83, 1.20)
	2021	-188 (-293, -83.5)	0.70 (0.55, 0.84)	-1620 (-1990, -1240)	0.70 (0.64, 0.76)	1.00 (0.80, 1.26)
Uzbekistan	2020	-629 (-761, -497)	0.74 (0.69, 0.79)	-2380 (-3920, -836)	0.81 (0.70, 0.92)	0.91 (0.78, 1.05)
	2021	-384 (-548, -221)	0.85 (0.78, 0.91)	86.2 (-1690, 1860)	1.01 (0.86, 1.15)	0.84 (0.72, 0.99)
Central Europe	2020	-1370 (-1550, -1180)	0.67 (0.64, 0.71)	-4870 (-5320, -4420)	0.70 (0.68, 0.72)	0.96 (0.90, 1.03)
	2021	-1250 (-1460, -1040)	0.68 (0.64, 0.72)	-3290 (-3800, -2790)	0.78 (0.75, 0.81)	0.87 (0.80, 0.93)
Albania	2020	-60.3 (-83.6, -36.9)	0.48 (0.34, 0.62)	-132 (-182, -81.3)	0.62 (0.50, 0.73)	0.78 (0.56, 1.10)
	2021	-60.2 (-89.3, -31)	0.50 (0.33, 0.67)	-93.4 (-154, -32.8)	0.73 (0.58, 0.88)	0.69 (0.45, 1.00)
Bosnia and Herzegovina	2020	-13.6 (-79.5, 52.2)	0.90 (0.42, 1.37)	-152 (-256, -49.4)	0.64 (0.45, 0.84)	1.39 (0.74, 2.49)
	2021	4.97 (-57.6, 67.5)	1.05 (0.44, 1.66)	-102 (-215, 11.2)	0.74 (0.49, 0.99)	1.42 (0.69, 2.76)
Bulgaria	2020	-15.8 (-30.9, -0.677)	0.93 (0.87, 1.00)	-227 (-282, -173)	0.75 (0.70, 0.80)	1.25 (1.13, 1.37)
	2021	-93.9 (-111, -77.1)	0.58 (0.53, 0.64)	-278 (-338, -218)	0.67 (0.61, 0.73)	0.87 (0.76, 1.00)
Croatia	2020	-38.4 (-53.8, -22.9)	0.65 (0.53, 0.76)	-80.1 (-101, -58.8)	0.58 (0.50, 0.67)	1.11 (0.90, 1.38)
	2021	-43.2 (-60, -26.3)	0.56 (0.44, 0.69)	-74.5 (-97.9, -51.2)	0.58 (0.48, 0.68)	0.98 (0.74, 1.28)

Czechia	2020	-4.49 (-20.4, 11.4)	0.95 (0.80, 1.11)	-94.8 (-136, -53.3)	0.75 (0.65, 0.84)	1.28 (1.04, 1.58)
	2021	-13.2 (-30.8, 4.37)	0.86 (0.68, 1.03)	-89 (-140, -38.3)	0.77 (0.65, 0.88)	1.12 (0.88, 1.45)
Hungary	2020	-21.8 (-40.7, -2.98)	0.82 (0.69, 0.96)	-66.5 (-119, -14.2)	0.82 (0.69, 0.95)	1.01 (0.80, 1.27)
	2021	-33.3 (-53.7, -12.9)	0.70 (0.55, 0.86)	-87.1 (-143, -30.9)	0.74 (0.59, 0.88)	0.95 (0.74, 1.31)
North Macedonia	2020	-10.8 (-16, -5.49)	0.65 (0.51, 0.79)	-35.5 (-48.8, -22.2)	0.78 (0.70, 0.85)	0.84 (0.66, 1.06)
	2021	-0.727 (-6.4, 4.95)	0.97 (0.77, 1.18)	-28.4 (-43.2, -13.6)	0.81 (0.72, 0.90)	1.21 (0.96, 1.56)
Poland	2020	-371 (-414, -328)	0.69 (0.66, 0.72)	-1120 (-1270, -976)	0.68 (0.65, 0.72)	1.01 (0.95, 1.08)
	2021	-332 (-380, -284)	0.70 (0.67, 0.74)	-557 (-723, -391)	0.83 (0.79, 0.88)	0.84 (0.78, 0.91)
Romania	2020	-604 (-759, -449)	0.65 (0.57, 0.72)	-2520 (-2920, -2130)	0.72 (0.68, 0.76)	0.90 (0.80, 1.03)
	2021	-446 (-623, -269)	0.73 (0.63, 0.82)	-1660 (-2110, -1220)	0.80 (0.76, 0.85)	0.90 (0.79, 1.04)
Serbia	2020	-219 (-270, -168)	0.33 (0.24, 0.42)	-387 (-433, -342)	0.46 (0.42, 0.51)	0.70 (0.53, 0.95)
	2021	-204 (-261, -146)	0.32 (0.21, 0.43)	-302 (-353, -252)	0.54 (0.48, 0.59)	0.60 (0.42, 0.84)
Slovakia	2020	-8.04 (-26.3, 10.3)	0.84 (0.51, 1.17)	-37.5 (-58.3, -16.6)	0.75 (0.63, 0.87)	1.13 (0.73, 1.72)
	2021	-28.6 (-48.5, -8.63)	0.39 (0.12, 0.65)	-14.3 (-36.9, 8.36)	0.89 (0.74, 1.05)	0.43 (0.20, 0.83)
Slovenia	2020	-0.444 (-7.53, 6.64)	0.99 (0.81, 1.17)	-15.7 (-22.6, -8.73)	0.71 (0.60, 0.82)	1.40 (1.09, 1.80)
	2021	-2.78 (-10.7, 5.1)	0.92 (0.71, 1.13)	-4.33 (-12.1, 3.45)	0.91 (0.77, 1.06)	1.01 (0.77, 1.35)
Eastern Europe	2020	-2230 (-3040, -1420)	0.74 (0.66, 0.82)	-20700 (-28900, -12400)	0.78 (0.70, 0.86)	0.95 (0.82, 1.11)
	2021	-1780 (-2750, -804)	0.79 (0.69, 0.89)	-16300 (-25700, -7000)	0.82 (0.72, 0.91)	0.97 (0.82, 1.15)
Belarus	2020	-126 (-169, -82.5)	0.61 (0.51, 0.72)	-289 (-401, -178)	0.82 (0.76, 0.89)	0.75 (0.61, 0.88)
	2021	-91.2 (-140, -42.8)	0.70 (0.57, 0.83)	-125 (-242, -8.9)	0.91 (0.84, 0.99)	0.77 (0.62, 0.95)
Estonia	2020	3 (-6.3, 12.3)	1.09 (0.81, 1.36)	-6.96 (-15.8, 1.91)	0.92 (0.83, 1.02)	1.18 (0.89, 1.51)
	2021	-3.53 (-14.2, 7.16)	0.89 (0.59, 1.20)	2.63 (-6.7, 12)	1.03 (0.91, 1.15)	0.87 (0.62, 1.21)
Latvia	2020					
	2021	-28.1 (-66.1, 9.82)	0.52 (0.06, 0.99)	-74 (-112, -35.9)	0.76 (0.65, 0.87)	0.69 (0.27, 1.72)
Lithuania	2020	-37.4 (-61.2, -13.5)	0.76 (0.63, 0.89)	-236 (-306, -165)	0.71 (0.64, 0.79)	1.07 (0.88, 1.30)
	2021	-40.2 (-66.9, -13.5)	0.73 (0.57, 0.88)	-224 (-302, -145)	0.71 (0.62, 0.79)	1.03 (0.80, 1.30)
Republic of Moldova	2020	-98.5 (-128, -69.6)	0.59 (0.50, 0.69)	-727 (-844, -609)	0.70 (0.66, 0.74)	0.85 (0.71, 1.00)
	2021	-52.1 (-86.5, -17.7)	0.78 (0.65, 0.91)	-312 (-442, -182)	0.86 (0.81, 0.91)	0.91 (0.76, 1.09)
Russian Federation	2020	-1310 (-2120, -504)	0.77 (0.65, 0.90)	-12900 (-21100, -4740)	0.81 (0.70, 0.92)	0.96 (0.79, 1.16)
	2021	-1280 (-2250, -318)	0.78 (0.63, 0.93)	-11100 (-20400, -1820)	0.82 (0.69, 0.96)	0.94 (0.74, 1.21)
Ukraine	2020	-600 (-686, -515)	0.70 (0.66, 0.74)	-6130 (-7070, -5190)	0.72 (0.69, 0.76)	0.97 (0.90, 1.04)
	2021	-278 (-376, -180)	0.86 (0.81, 0.90)	-4520 (-5590, -3440)	0.79 (0.74, 0.83)	1.09 (1.01, 1.18)
High-income	2020	-5270 (-6140, -4410)	0.85 (0.82, 0.87)	-7640 (-9350, -5940)	0.89 (0.87, 0.91)	0.95 (0.92, 0.99)
	2021	-5060 (-6110, -4010)	0.85 (0.82, 0.88)	-5360 (-7470, -3250)	0.92 (0.89, 0.95)	0.92 (0.88, 0.96)
High-income Asia Pacific	2020	-3180 (-4000, -2350)	0.87 (0.83, 0.90)	-1880 (-2460, -1300)	0.91 (0.88, 0.94)	0.95 (0.91, 1.00)
	2021	-3830 (-4830, -2830)	0.84 (0.80, 0.88)	-2130 (-2760, -1490)	0.89 (0.86, 0.92)	0.94 (0.89, 1.00)
Brunei Darussalam	2020	15.3 (8.09, 22.5)	1.26 (1.12, 1.40)	43.5 (17.8, 69.1)	1.21 (1.07, 1.34)	1.05 (0.89, 1.23)
	2021	-12.3 (-21.5, -3.04)	0.80 (0.67, 0.94)	-18.3 (-50.5, 13.9)	0.92 (0.78, 1.06)	0.88 (0.70, 1.11)
Japan	2020	-487 (-628, -346)	0.95 (0.93, 0.96)	-300 (-590, -10.9)	0.94 (0.88, 1.00)	1.01 (0.95, 1.08)
	2021	-707 (-865, -549)	0.92 (0.90, 0.94)	-538 (-866, -209)	0.88 (0.81, 0.95)	1.04 (0.96, 1.12)
Republic of Korea	2020	-2700 (-3510, -1890)	0.81 (0.76, 0.86)	-1770 (-2240, -1290)	0.88 (0.84, 0.91)	0.92 (0.86, 0.99)

	2021	-3090 (-4070, -2110)	0.78 (0.72, 0.84)	-1830 (-2340, -1310)	0.86 (0.82, 0.89)	0.91 (0.84, 0.99)
Singapore	2020	-7.99 (-91.2, 75.2)	0.99 (0.86, 1.12)	147 (-5.88, 299)	1.09 (0.99, 1.18)	0.91 (0.77, 1.05)
	2021	-18.9 (-121, 83.5)	0.97 (0.81, 1.13)	255 (71.4, 438)	1.15 (1.03, 1.27)	0.84 (0.70, 1.02)
Australasia	2020	-5.59 (-36.2, 25)	0.98 (0.89, 1.07)	85.2 (20.7, 150)	1.05 (1.01, 1.10)	0.93 (0.84, 1.03)
	2021	-50.3 (-89, -11.5)	0.85 (0.75, 0.96)	-104 (-184, -23.7)	0.93 (0.89, 0.98)	0.91 (0.79, 1.04)
Australia	2020	-11.7 (-40.1, 16.6)	0.96 (0.86, 1.06)	77.4 (21.2, 134)	1.06 (1.02, 1.11)	0.90 (0.80, 1.01)
	2021	-38.1 (-74.1, -2.05)	0.87 (0.75, 0.98)	-97.4 (-167, -27.7)	0.93 (0.87, 0.98)	0.94 (0.81, 1.09)
New Zealand	2020	6.15 (-5.36, 17.7)	1.11 (0.90, 1.32)	7.85 (-23.8, 39.5)	1.03 (0.91, 1.14)	1.08 (0.86, 1.33)
	2021	-12.2 (-26.4, 2.02)	0.79 (0.58, 1.01)	-6.27 (-45.5, 32.9)	0.98 (0.84, 1.11)	0.81 (0.61, 1.10)
Western Europe	2020	-883 (-1100, -671)	0.83 (0.79, 0.86)	-2920 (-3910, -1940)	0.88 (0.84, 0.92)	0.94 (0.88, 1.00)
	2021	-528 (-788, -267)	0.89 (0.84, 0.94)	-2470 (-3680, -1250)	0.89 (0.84, 0.94)	1.00 (0.92, 1.08)
Austria	2020	1.06 (-12.7, 14.8)	1.01 (0.86, 1.17)	-68.3 (-122, -14.2)	0.82 (0.70, 0.95)	1.23 (0.99, 1.51)
	2021	1.8 (-13.8, 17.4)	1.02 (0.84, 1.21)	-41.7 (-104, 20.9)	0.89 (0.73, 1.05)	1.15 (0.89, 1.47)
Belgium	2020	-4.03 (-31.1, 23)	0.97 (0.75, 1.19)	-111 (-150, -71)	0.87 (0.82, 0.91)	1.11 (0.89, 1.38)
	2021	-18.6 (-50.3, 13)	0.84 (0.60, 1.09)	-18.9 (-67.3, 29.5)	0.98 (0.92, 1.03)	0.86 (0.63, 1.14)
Denmark	2020	-3.94 (-11.3, 3.39)	0.86 (0.62, 1.10)	-27.2 (-57.3, 2.86)	0.88 (0.75, 1.00)	0.98 (0.71, 1.33)
	2021	2.11 (-6.71, 10.9)	1.08 (0.75, 1.40)	-48.9 (-83.6, -14.1)	0.78 (0.64, 0.92)	1.39 (1.00, 1.96)
Finland	2020	-1.59 (-21, 17.9)	0.98 (0.76, 1.20)	-38 (-56.8, -19.2)	0.71 (0.59, 0.83)	1.37 (1.01, 1.82)
	2021	-6.18 (-28.3, 16)	0.93 (0.67, 1.18)	-25.7 (-48.1, -3.33)	0.80 (0.65, 0.96)	1.15 (0.83, 1.63)
France	2020	-89.8 (-149, -30.1)	0.89 (0.82, 0.96)	-141 (-508, 227)	0.97 (0.88, 1.05)	0.92 (0.82, 1.04)
	2021	-32.5 (-102, 37.1)	0.96 (0.87, 1.04)	-740 (-1200, -282)	0.83 (0.74, 0.93)	1.15 (0.99, 1.33)
Germany	2020	-12.4 (-79.1, 54.3)	0.98 (0.90, 1.07)	-531 (-829, -234)	0.87 (0.81, 0.94)	1.13 (1.00, 1.25)
	2021	-73.4 (-154, 7.52)	0.90 (0.80, 1.00)	-610 (-981, -239)	0.85 (0.77, 0.94)	1.06 (0.92, 1.22)
Greece	2020	-29.5 (-45.4, -13.7)	0.72 (0.59, 0.85)	18.6 (-56.6, 93.8)	1.06 (0.82, 1.29)	0.68 (0.52, 0.92)
	2021	-71.9 (-90.1, -53.7)	0.29 (0.19, 0.38)	-132 (-223, -41.5)	0.60 (0.38, 0.81)	0.48 (0.29, 0.78)
Ireland	2020	-20.5 (-31.1, -9.88)	0.68 (0.54, 0.81)	1.91 (-27.8, 31.7)	1.01 (0.86, 1.16)	0.67 (0.52, 0.85)
	2021	-35.8 (-48.9, -22.7)	0.45 (0.31, 0.58)	2.63 (-31.2, 36.5)	1.01 (0.83, 1.20)	0.44 (0.32, 0.63)
Israel	2020	-22.3 (-51.6, 7)	0.62 (0.22, 1.01)	-38 (-70.1, -5.9)	0.77 (0.60, 0.94)	0.80 (0.42, 1.60)
	2021	-13.2 (-49.1, 22.7)	0.78 (0.24, 1.31)	-10.7 (-46.2, 24.8)	0.93 (0.71, 1.15)	0.84 (0.40, 1.82)
Italy	2020	-259 (-355, -163)	0.61 (0.50, 0.72)	-1030 (-1580, -471)	0.66 (0.51, 0.81)	0.92 (0.70, 1.23)
	2021	-258 (-393, -124)	0.63 (0.47, 0.78)	-838 (-1580, -94.1)	0.72 (0.51, 0.93)	0.87 (0.60, 1.26)
Netherlands	2020	-13.9 (-28.6, 0.891)	0.87 (0.73, 1.00)	-106 (-150, -62.7)	0.85 (0.79, 0.90)	1.02 (0.87, 1.20)
	2021	26.3 (9.26, 43.3)	1.27 (1.07, 1.46)	-84.2 (-137, -31.8)	0.88 (0.81, 0.95)	1.44 (1.20, 1.73)
Norway	2020	-3.67 (-10.4, 3.11)	0.79 (0.45, 1.13)	4.15 (-11.5, 19.8)	1.03 (0.92, 1.14)	0.77 (0.50, 1.15)
	2021	-0.135 (-8.02, 7.75)	0.99 (0.53, 1.45)	10.1 (-6.21, 26.5)	1.08 (0.95, 1.22)	0.92 (0.57, 1.52)
Portugal	2020	-75.3 (-90.4, -60.2)	0.81 (0.78, 0.84)	-62.8 (-233, 107)	0.95 (0.82, 1.08)	0.85 (0.74, 0.98)
	2021	23.9 (6.45, 41.4)	1.06 (1.02, 1.11)	-47.3 (-241, 146)	0.96 (0.80, 1.12)	1.11 (0.93, 1.31)
Spain	2020	-258 (-408, -108)	0.71 (0.57, 0.85)	-755 (-1180, -335)	0.77 (0.65, 0.88)	0.93 (0.73, 1.18)
	2021	-120 (-297, 56.4)	0.86 (0.68, 1.05)	-361 (-852, 130)	0.89 (0.74, 1.03)	0.98 (0.75, 1.29)
Sweden	2020	-9.76 (-16.2, -3.37)	0.82 (0.72, 0.93)	-120 (-258, 18.6)	0.72 (0.45, 1.00)	1.13 (0.77, 1.69)
	2021	12.8 (5.38, 20.3)	1.24 (1.09, 1.40)	-94.2 (-251, 62.4)	0.77 (0.44, 1.11)	1.61 (1.03, 2.52)

Switzerland	2020	-26.8 (-39.9, -13.8)	0.65 (0.51, 0.79)	-58.7 (-158, 40.7)	0.85 (0.62, 1.08)	0.76 (0.54, 1.09)
	2021	-19.8 (-35.7, -3.9)	0.75 (0.57, 0.92)	-61.5 (-180, 56.6)	0.84 (0.56, 1.12)	0.89 (0.59, 1.30)
United Kingdom	2020	-54.2 (-98.6, -9.87)	0.92 (0.86, 0.98)	135 (-295, 565)	1.04 (0.92, 1.15)	0.89 (0.77, 1.00)
	2021	55.8 (6.17, 105)	1.09 (1.01, 1.17)	636 (157, 1120)	1.18 (1.03, 1.33)	0.92 (0.80, 1.06)
Southern Latin America	2020	-576 (-707, -445)	0.71 (0.65, 0.76)	-1910 (-3120, -686)	0.87 (0.79, 0.95)	0.81 (0.72, 0.92)
	2021	-359 (-525, -194)	0.83 (0.75, 0.90)	-59.9 (-1610, 1490)	1.00 (0.90, 1.10)	0.83 (0.73, 0.95)
Argentina	2020	-371 (-482, -259)	0.69 (0.62, 0.77)	-1420 (-2620, -216)	0.87 (0.77, 0.97)	0.79 (0.68, 0.93)
	2021	-204 (-346, -61.9)	0.84 (0.74, 0.94)	476 (-1050, 2000)	1.04 (0.91, 1.17)	0.81 (0.68, 0.96)
Chile	2020	-202 (-266, -139)	0.67 (0.59, 0.76)	-406 (-602, -209)	0.84 (0.77, 0.91)	0.80 (0.68, 0.92)
	2021	-123 (-201, -44.3)	0.81 (0.70, 0.92)	-435 (-686, -185)	0.84 (0.76, 0.92)	0.96 (0.81, 1.13)
Uruguay	2020	-3.28 (-28.7, 22.1)	0.98 (0.80, 1.15)	-81.6 (-138, -25.7)	0.91 (0.85, 0.97)	1.07 (0.89, 1.30)
	2021	-32.7 (-65.2, -0.0793)	0.78 (0.59, 0.97)	-101 (-170, -31.4)	0.89 (0.82, 0.96)	0.88 (0.67, 1.12)
High-income North America	2020	-630 (-694, -565)	0.77 (0.75, 0.79)	-1020 (-1350, -689)	0.87 (0.83, 0.91)	0.89 (0.84, 0.94)
	2021	-294 (-372, -215)	0.90 (0.87, 0.92)	-605 (-1000, -206)	0.92 (0.87, 0.97)	0.97 (0.91, 1.03)
Canada	2020	-96.6 (-125, -67.9)	0.80 (0.75, 0.86)	-184 (-345, -22.9)	0.88 (0.78, 0.98)	0.91 (0.81, 1.04)
	2021	-62.6 (-98.3, -26.8)	0.88 (0.81, 0.94)	-233 (-436, -30)	0.86 (0.74, 0.97)	1.03 (0.87, 1.20)
United States of America	2020	-533 (-591, -476)	0.77 (0.75, 0.79)	-837 (-1130, -547)	0.87 (0.83, 0.91)	0.88 (0.83, 0.93)
	2021	-231 (-301, -161)	0.90 (0.87, 0.93)	-372 (-715, -28.4)	0.94 (0.89, 0.99)	0.96 (0.90, 1.02)
Latin America and Caribbean	2020	-6980 (-7530, -6420)	0.74 (0.72, 0.75)	-30100 (-39500, -20600)	0.85 (0.81, 0.90)	0.86 (0.82, 0.91)
	2021	-4470 (-5170, -3770)	0.84 (0.81, 0.86)	-25200 (-37000, -13300)	0.88 (0.83, 0.93)	0.95 (0.88, 1.01)
Caribbean	2020	-247 (-443, -50.1)	0.81 (0.68, 0.95)	-2970 (-4380, -1560)	0.83 (0.76, 0.90)	0.98 (0.81, 1.17)
	2021	-232 (-492, 28.6)	0.83 (0.65, 1.00)	-1700 (-3430, 32.2)	0.90 (0.80, 1.00)	0.92 (0.72, 1.16)
Belize	2020	-3.34 (-7.65, 0.985)	0.64 (0.27, 1.01)	-14.4 (-47.7, 18.9)	0.85 (0.52, 1.17)	0.76 (0.37, 1.55)
	2021	-3.59 (-8.34, 1.16)	0.58 (0.16, 1.00)	-20.9 (-61.7, 20)	0.78 (0.41, 1.16)	0.74 (0.33, 1.83)
Cuba	2020	-27.3 (-53.1, -1.43)	0.74 (0.53, 0.95)	-32.7 (-75.5, 9.99)	0.94 (0.87, 1.02)	0.78 (0.58, 1.03)
	2021	-24.1 (-53.9, 5.68)	0.76 (0.50, 1.02)	-100 (-151, -49.7)	0.82 (0.74, 0.90)	0.93 (0.64, 1.33)
Dominican Republic	2020	-180 (-366, 6.05)	0.60 (0.27, 0.92)	-1450 (-2630, -266)	0.64 (0.41, 0.88)	0.93 (0.48, 1.84)
	2021	-86.1 (-335, 163)	0.82 (0.36, 1.29)	-986 (-2490, 515)	0.77 (0.46, 1.08)	1.07 (0.55, 2.09)
Guyana	2020	-15.1 (-26.1, -4.17)	0.57 (0.33, 0.80)	-59.2 (-109, -9.25)	0.87 (0.77, 0.97)	0.65 (0.43, 0.98)
	2021	8.97 (-3.37, 21.3)	1.27 (0.85, 1.69)	-43.8 (-101, 13.8)	0.90 (0.78, 1.02)	1.41 (0.98, 1.97)
Haiti	2020	6.92 (-48.2, 62)	1.01 (0.93, 1.10)	-1380 (-2130, -626)	0.89 (0.83, 0.94)	1.14 (1.03, 1.28)
	2021	-81 (-145, -16.7)	0.87 (0.78, 0.97)	-483 (-1340, 377)	0.96 (0.88, 1.03)	0.91 (0.80, 1.03)
Jamaica	2020	-10.9 (-14.1, -7.81)	0.22 (0.11, 0.32)	-11.8 (-36.7, 13.2)	0.83 (0.50, 1.16)	0.26 (0.14, 0.46)
	2021	-8.63 (-13.3, -4.01)	0.48 (0.29, 0.67)	-17.7 (-46.4, 10.9)	0.73 (0.37, 1.10)	0.66 (0.35, 1.22)
Suriname	2020	-16.4 (-23.7, -9.15)	0.23 (0.07, 0.40)	-30 (-64.5, 4.49)	0.77 (0.54, 1.00)	0.30 (0.13, 0.69)
	2021	-20 (-31.5, -8.51)	0.26 (0.04, 0.48)	-41.7 (-83.1, -0.246)	0.68 (0.42, 0.94)	0.38 (0.15, 0.94)
Trinidad and Tobago	2020	-0.574 (-12.1, 10.9)	0.98 (0.68, 1.29)	6.91 (-26.6, 40.4)	1.04 (0.85, 1.23)	0.95 (0.67, 1.36)
	2021	-17.1 (-31.6, -2.57)	0.56 (0.28, 0.84)	-8.21 (-47.3, 30.9)	0.95 (0.73, 1.17)	0.59 (0.36, 1.03)
Andean Latin America	2020	-2310 (-2630, -1990)	0.64 (0.60, 0.68)	-8850 (-11500, -6200)	0.79 (0.74, 0.85)	0.81 (0.73, 0.88)
	2021	-1520 (-1920, -1120)	0.77 (0.72, 0.82)	-7790 (-11100, -4460)	0.82 (0.76, 0.89)	0.93 (0.83, 1.04)
	2020	-382 (-471, -292)	0.70 (0.64, 0.76)	-913 (-1230, -597)	0.85 (0.80, 0.90)	0.83 (0.75, 0.91)

Bolivia (Plurinational State of)	2021	-125 (-231, -18.8)	0.90 (0.82, 0.98)	42.5 (-329, 414)	1.01 (0.94, 1.07)	0.89 (0.80, 1.00)
Ecuador	2020	-262 (-336, -188)	0.68 (0.60, 0.75)	-879 (-1280, -480)	0.84 (0.78, 0.91)	0.81 (0.71, 0.92)
	2021	-297 (-389, -205)	0.65 (0.56, 0.73)	-715 (-1210, -217)	0.88 (0.80, 0.96)	0.74 (0.62, 0.87)
Peru	2020	-1670 (-1970, -1370)	0.61 (0.56, 0.67)	-7060 (-9670, -4460)	0.78 (0.70, 0.85)	0.79 (0.69, 0.90)
	2021	-1100 (-1470, -725)	0.75 (0.68, 0.83)	-7110 (-10400, -3840)	0.78 (0.69, 0.87)	0.97 (0.83, 1.12)
Central Latin America	2020	-3100 (-3320, -2870)	0.68 (0.66, 0.70)	-11700 (-14000, -9360)	0.80 (0.77, 0.84)	0.84 (0.80, 0.89)
	2021	-2050 (-2330, -1770)	0.79 (0.76, 0.82)	-9260 (-12300, -6180)	0.85 (0.80, 0.90)	0.93 (0.88, 0.99)
Colombia	2020	-824 (-943, -705)	0.74 (0.70, 0.77)	-285 (-727, 157)	0.98 (0.94, 1.01)	0.76 (0.71, 0.80)
	2021	-741 (-892, -590)	0.77 (0.73, 0.81)	904 (358, 1450)	1.08 (1.03, 1.13)	0.72 (0.67, 0.77)
Costa Rica	2020					
	2021	-22.8 (-40.8, -4.81)	0.68 (0.47, 0.89)	9.25 (-46.7, 65.2)	1.03 (0.85, 1.20)	0.66 (0.45, 0.92)
El Salvador	2020	-125 (-197, -53.6)	0.59 (0.41, 0.77)	-1650 (-2770, -520)	0.53 (0.30, 0.76)	1.12 (0.64, 1.91)
	2021	-54.7 (-138, 28.9)	0.82 (0.56, 1.07)	-2160 (-3670, -653)	0.44 (0.18, 0.70)	1.86 (0.91, 3.74)
Guatemala	2020	-106 (-168, -44.5)	0.72 (0.58, 0.86)	-1140 (-1790, -483)	0.71 (0.57, 0.85)	1.01 (0.78, 1.33)
	2021	-75.9 (-152, -0.186)	0.80 (0.63, 0.98)	-1160 (-2010, -299)	0.73 (0.55, 0.90)	1.10 (0.80, 1.55)
Honduras	2020	-192 (-262, -121)	0.58 (0.47, 0.70)	-405 (-488, -323)	0.82 (0.79, 0.85)	0.71 (0.59, 0.86)
	2021	-130 (-215, -46.2)	0.71 (0.56, 0.87)	-217 (-315, -120)	0.90 (0.86, 0.94)	0.79 (0.64, 1.01)
Mexico	2020	-1320 (-1400, -1240)	0.63 (0.61, 0.65)	-4890 (-6000, -3780)	0.78 (0.73, 0.82)	0.81 (0.76, 0.87)
	2021	-629 (-727, -531)	0.83 (0.80, 0.85)	-1990 (-3370, -608)	0.91 (0.85, 0.97)	0.90 (0.84, 0.97)
Nicaragua	2020	-102 (-167, -36.2)	0.66 (0.48, 0.84)	-636 (-1150, -120)	0.73 (0.54, 0.92)	0.90 (0.62, 1.35)
	2021	-123 (-211, -35.6)	0.62 (0.41, 0.83)	-872 (-1550, -197)	0.65 (0.43, 0.87)	0.95 (0.57, 1.57)
Panama	2020	-75 (-119, -31.1)	0.60 (0.42, 0.78)	-413 (-777, -47.8)	0.71 (0.50, 0.93)	0.84 (0.56, 1.29)
	2021	-51.9 (-105, 1.23)	0.73 (0.49, 0.96)	-114 (-553, 324)	0.92 (0.63, 1.21)	0.79 (0.48, 1.26)
Venezuela (Bolivarian Republic of)	2020	-277 (-384, -169)	0.76 (0.68, 0.84)	-1950 (-3340, -568)	0.83 (0.72, 0.94)	0.91 (0.77, 1.10)
	2021	-219 (-351, -87.9)	0.81 (0.71, 0.91)	-3670 (-5580, -1760)	0.72 (0.59, 0.84)	1.13 (0.91, 1.38)
Tropical Latin America	2020	-1320 (-1660, -982)	0.86 (0.82, 0.89)	-6570 (-15200, 2090)	0.92 (0.83, 1.02)	0.93 (0.83, 1.04)
	2021	-669 (-1090, -247)	0.93 (0.89, 0.97)	-6420 (-17200, 4400)	0.93 (0.81, 1.04)	1.00 (0.88, 1.15)
Brazil	2020	-1230 (-1560, -893)	0.86 (0.83, 0.90)	-6250 (-14900, 2410)	0.93 (0.83, 1.02)	0.93 (0.83, 1.05)
	2021	-594 (-1010, -177)	0.93 (0.89, 0.98)	-6340 (-17100, 4470)	0.93 (0.81, 1.05)	1.01 (0.88, 1.16)
Paraguay	2020	-92.5 (-145, -40.2)	0.74 (0.61, 0.87)	-320 (-604, -35.8)	0.88 (0.79, 0.98)	0.84 (0.68, 1.03)
	2021	-74.9 (-141, -9.11)	0.80 (0.64, 0.96)	-78.8 (-442, 284)	0.97 (0.85, 1.10)	0.82 (0.66, 1.06)
North Africa and Middle East	2020	-3900 (-4290, -3510)	0.78 (0.76, 0.80)	-19500 (-23100, -15800)	0.86 (0.83, 0.88)	0.91 (0.88, 0.94)
	2021	-2870 (-3360, -2380)	0.84 (0.81, 0.86)	-16100 (-21200, -11100)	0.88 (0.85, 0.92)	0.95 (0.90, 1.00)
North Africa and Middle East	2020	-3900 (-4290, -3510)	0.78 (0.76, 0.80)	-19500 (-23100, -15800)	0.86 (0.83, 0.88)	0.91 (0.87, 0.94)
	2021	-2870 (-3360, -2380)	0.84 (0.81, 0.86)	-16100 (-21200, -11100)	0.88 (0.85, 0.92)	0.95 (0.91, 1.00)
Algeria	2020	-234 (-262, -206)	0.69 (0.66, 0.72)	-877 (-1130, -623)	0.80 (0.74, 0.85)	0.87 (0.81, 0.94)
	2021	-135 (-168, -102)	0.82 (0.78, 0.86)	-236 (-519, 46.7)	0.94 (0.87, 1.01)	0.87 (0.80, 0.95)
Bahrain	2020	-0.184 (-3.25, 2.88)	0.98 (0.65, 1.31)	5.72 (-31.7, 43.2)	1.03 (0.83, 1.23)	0.95 (0.63, 1.43)
	2021	-1.4 (-5.14, 2.34)	0.83 (0.43, 1.24)	-14.6 (-69.7, 40.5)	0.93 (0.67, 1.19)	0.90 (0.52, 1.63)
Egypt	2020	-295 (-500, -89.5)	0.62 (0.42, 0.83)	-1150 (-1660, -650)	0.85 (0.79, 0.91)	0.73 (0.51, 1.03)

	2021	-327 (-592, -62.5)	0.61 (0.36, 0.85)	-1480 (-2090, -864)	0.81 (0.74, 0.88)	0.75 (0.50, 1.14)
Iran (Islamic Republic of)	2020	-895 (-1030, -765)	0.66 (0.62, 0.70)	-1440 (-1800, -1080)	0.75 (0.69, 0.80)	0.88 (0.79, 0.97)
	2021	-692 (-845, -539)	0.73 (0.68, 0.78)	-719 (-1130, -309)	0.87 (0.80, 0.94)	0.84 (0.76, 0.94)
Iraq	2020	-268 (-347, -188)	0.72 (0.65, 0.79)	-1170 (-1480, -866)	0.79 (0.74, 0.84)	0.92 (0.81, 1.02)
	2021	-200 (-291, -110)	0.78 (0.69, 0.87)	-124 (-475, 226)	0.98 (0.91, 1.04)	0.80 (0.70, 0.91)
Jordan	2020	-17.3 (-30.8, -3.76)	0.39 (0.09, 0.69)	-257 (-347, -168)	0.48 (0.35, 0.60)	0.82 (0.36, 1.81)
	2021	-12.8 (-28.2, 2.54)	0.52 (0.11, 0.94)	-312 (-426, -198)	0.40 (0.26, 0.53)	1.32 (0.58, 3.13)
Kuwait	2020	0.888 (-4.29, 6.07)	1.03 (0.84, 1.22)	-200 (-347, -53.8)	0.77 (0.62, 0.92)	1.34 (1.02, 1.76)
	2021	-3.24 (-9.74, 3.25)	0.89 (0.68, 1.10)	-169 (-349, 11.9)	0.81 (0.62, 0.99)	1.10 (0.81, 1.51)
Lebanon	2020	2.59 (-9.16, 14.3)	1.06 (0.76, 1.36)	12 (-165, 189)	1.02 (0.75, 1.28)	1.05 (0.71, 1.52)
	2021	2.2 (-11.4, 15.8)	1.06 (0.70, 1.42)	-190 (-405, 24.1)	0.72 (0.45, 0.99)	1.47 (0.92, 2.41)
Libya	2020	-27.4 (-61.4, 6.59)	0.82 (0.62, 1.02)	-342 (-986, 302)	0.83 (0.53, 1.12)	0.99 (0.64, 1.58)
	2021	-40.3 (-84.6, 4)	0.75 (0.52, 0.99)	-369 (-1240, 504)	0.83 (0.47, 1.20)	0.91 (0.51, 1.55)
Morocco	2020	-354 (-558, -150)	0.88 (0.82, 0.95)	-2880 (-4370, -1400)	0.90 (0.85, 0.95)	0.98 (0.89, 1.07)
	2021	-251 (-507, 5.5)	0.92 (0.84, 1.00)	-3230 (-5050, -1410)	0.89 (0.83, 0.95)	1.03 (0.93, 1.14)
Oman	2020	-6.58 (-21.8, 8.6)	0.84 (0.50, 1.18)	10.7 (-73.5, 95)	1.04 (0.72, 1.36)	0.81 (0.48, 1.38)
	2021	-5.53 (-24.3, 13.2)	0.87 (0.45, 1.29)	-65 (-164, 33.9)	0.75 (0.42, 1.08)	1.15 (0.60, 2.30)
Qatar	2020	-9.02 (-12.3, -5.76)	0.53 (0.40, 0.65)	-305 (-376, -235)	0.74 (0.68, 0.79)	0.71 (0.56, 0.91)
	2021	-2.93 (-9.09, 3.22)	0.89 (0.66, 1.11)	-469 (-588, -350)	0.67 (0.60, 0.74)	1.32 (1.02, 1.72)
Saudi Arabia	2020	-73.1 (-108, -38)	0.73 (0.62, 0.84)	-214 (-410, -18.7)	0.91 (0.84, 0.99)	0.80 (0.68, 0.94)
	2021	-42.1 (-84.8, 0.573)	0.85 (0.71, 0.99)	-23.1 (-249, 202)	0.99 (0.90, 1.09)	0.86 (0.70, 1.04)
Syrian Arab Republic	2020	-46.2 (-106, 13.6)	0.79 (0.54, 1.03)	-0.148 (-542, 542)	1.00 (0.81, 1.19)	0.79 (0.54, 1.13)
	2021	-25.8 (-98.1, 46.5)	0.88 (0.57, 1.19)	622 (-60.7, 1300)	1.21 (0.96, 1.46)	0.73 (0.48, 1.08)
Tunisia	2020	-71.1 (-98.6, -43.6)	0.84 (0.79, 0.90)	-250 (-1610, 1110)	0.91 (0.46, 1.36)	0.92 (0.56, 1.55)
	2021	-109 (-143, -74.7)	0.77 (0.70, 0.83)	-696 (-2380, 993)	0.77 (0.27, 1.26)	1.00 (0.52, 1.92)
Türkiye	2020	-705 (-833, -577)	0.70 (0.65, 0.74)	-952 (-1500, -402)	0.89 (0.84, 0.95)	0.78 (0.71, 0.86)
	2021	-600 (-755, -444)	0.75 (0.69, 0.80)	-511 (-1150, 125)	0.94 (0.87, 1.01)	0.79 (0.71, 0.88)
United Arab Emirates	2020	-1.03 (-8.11, 6.05)	0.94 (0.56, 1.32)	-19.8 (-35.1, -4.43)	0.68 (0.48, 0.88)	1.39 (0.85, 2.31)
	2021	-7.92 (-17.4, 1.54)	0.60 (0.23, 0.97)	-8.58 (-27.3, 10.2)	0.86 (0.58, 1.14)	0.70 (0.35, 1.43)
Yemen	2020	-77.2 (-153, -1.48)	0.91 (0.83, 0.99)	-791 (-1500, -83.3)	0.91 (0.83, 0.99)	1.00 (0.89, 1.14)
	2021	7.46 (-84.7, 99.6)	1.01 (0.91, 1.11)	-303 (-1150, 544)	0.97 (0.87, 1.06)	1.04 (0.89, 1.20)
South Asia	2020	-103000 (-124000, -80600)	0.68 (0.63, 0.74)	-597000 (-940000, -254000)	0.78 (0.67, 0.89)	0.87 (0.74, 1.02)
	2021	-67100 (-96400, -37700)	0.81 (0.73, 0.88)	-320000 (-777000, 138000)	0.89 (0.74, 1.04)	0.91 (0.75, 1.10)
South Asia	2020	-103000 (-124000, -80600)	0.68 (0.63, 0.74)	-597000 (-940000, -254000)	0.78 (0.67, 0.89)	0.87 (0.74, 1.02)
	2021	-67100 (-96400, -37700)	0.81 (0.73, 0.88)	-320000 (-777000, 138000)	0.89 (0.74, 1.04)	0.91 (0.75, 1.10)
Afghanistan	2020	-824 (-932, -715)	0.83 (0.81, 0.85)	-8640 (-11300, -6000)	0.83 (0.78, 0.88)	1.00 (0.94, 1.07)
	2021	-423 (-586, -260)	0.92 (0.89, 0.95)	-7840 (-11900, -3780)	0.85 (0.78, 0.92)	1.07 (0.98, 1.17)
Bangladesh	2020	-16400 (-19400, -13300)	0.71 (0.66, 0.75)	-66400 (-73200, -59700)	0.74 (0.72, 0.76)	0.95 (0.89, 1.03)
	2021	-9270 (-13500, -5040)	0.85 (0.79, 0.91)	-22800 (-31600, -14000)	0.92 (0.89, 0.95)	0.93 (0.86, 1.00)
Bhutan	2020	-11.8 (-33.8, 10.2)	0.85 (0.59, 1.11)	20 (-111, 151)	1.02 (0.86, 1.18)	0.83 (0.56, 1.18)
	2021	3.58 (-22.7, 29.9)	1.05 (0.70, 1.39)	-26.4 (-179, 126)	0.97 (0.78, 1.15)	1.08 (0.75, 1.56)

India	2020	-70900 (-92100, -49800)	0.68 (0.60, 0.76)	-453000 (-794000, -112000)	0.79 (0.65, 0.93)	0.86 (0.70, 1.05)
	2021	-48800 (-77100, -20500)	0.79 (0.69, 0.90)	-276000 (-731000, 179000)	0.88 (0.69, 1.07)	0.90 (0.71, 1.12)
Nepal	2020	-1610 (-2030, -1180)	0.74 (0.68, 0.80)	-3490 (-4170, -2810)	0.86 (0.84, 0.89)	0.86 (0.79, 0.93)
	2021	-1850 (-2450, -1250)	0.73 (0.65, 0.80)	-2200 (-3060, -1340)	0.91 (0.88, 0.95)	0.80 (0.71, 0.89)
Pakistan	2020	-13600 (-18500, -8710)	0.68 (0.59, 0.78)	-73200 (-109000, -37400)	0.77 (0.67, 0.87)	0.89 (0.73, 1.07)
	2021	-7140 (-13500, -734)	0.84 (0.71, 0.97)	-18500 (-63200, 26200)	0.94 (0.81, 1.08)	0.89 (0.73, 1.08)
Sub-Saharan Africa	2020	-20400 (-26000, -14700)	0.78 (0.72, 0.83)	-60800 (-125000, 3630)	0.95 (0.89, 1.00)	0.82 (0.75, 0.90)
	2021	-13000 (-21600, -4380)	0.87 (0.80, 0.95)	-59900 (-153000, 33000)	0.95 (0.88, 1.02)	0.92 (0.80, 1.03)
Central Sub-Saharan Africa	2020	-5850 (-10700, -991)	0.72 (0.52, 0.92)	-58200 (-116000, -182)	0.79 (0.60, 0.98)	0.91 (0.64, 1.28)
	2021	-9940 (-17600, -2230)	0.62 (0.39, 0.85)	-99600 (-185000, -13700)	0.70 (0.48, 0.92)	0.89 (0.55, 1.42)
Central African Republic	2020	-117 (-236, 1.58)	0.81 (0.63, 0.98)	-3170 (-5760, -578)	0.79 (0.64, 0.94)	1.02 (0.77, 1.35)
	2021	-190 (-381, 0.601)	0.75 (0.54, 0.97)	-6010 (-9910, -2110)	0.68 (0.50, 0.85)	1.12 (0.75, 1.64)
Congo	2020	23.6 (-82, 129)	1.03 (0.90, 1.16)	-1250 (-2520, 14.9)	0.89 (0.79, 1.00)	1.15 (0.98, 1.36)
	2021	75.7 (-70.2, 222)	1.08 (0.91, 1.25)	-1260 (-2990, 470)	0.90 (0.76, 1.03)	1.21 (0.97, 1.48)
Democratic Republic of the Congo	2020	-5570 (-10400, -719)	0.70 (0.48, 0.92)	-53300 (-111000, 4620)	0.78 (0.57, 0.99)	0.90 (0.57, 1.38)
	2021	-9660 (-17400, -1970)	0.60 (0.35, 0.85)	-92300 (-178000, -6590)	0.68 (0.44, 0.93)	0.88 (0.50, 1.52)
Equatorial Guinea	2020	-44 (-87.5, -0.447)	0.56 (0.23, 0.88)	-50.7 (-326, 224)	0.97 (0.81, 1.13)	0.57 (0.32, 1.08)
	2021	-34.2 (-98.9, 30.5)	0.71 (0.24, 1.17)	215 (-151, 582)	1.12 (0.90, 1.34)	0.63 (0.32, 1.29)
Gabon	2020	-144 (-417, 128)	0.55 (0.00, 1.18)	-408 (-876, 60.9)	0.92 (0.83, 1.01)	0.60 (0.18, 1.93)
	2021	-129 (-458, 199)	0.60 (0.00, 1.38)	-204 (-780, 372)	0.96 (0.85, 1.07)	0.63 (0.16, 2.24)
Eastern Sub-Saharan Africa	2020	-6520 (-8610, -4420)	0.82 (0.76, 0.87)	-24800 (-42300, -7250)	0.94 (0.90, 0.98)	0.87 (0.80, 0.93)
	2021	-7440 (-10400, -4480)	0.81 (0.74, 0.88)	-31700 (-56400, -7020)	0.93 (0.87, 0.98)	0.87 (0.79, 0.97)
Burundi	2020	-84.3 (-165, -3.61)	0.87 (0.75, 0.99)	-116 (-852, 620)	0.98 (0.87, 1.09)	0.88 (0.74, 1.04)
	2021	6.87 (-98.6, 112)	1.01 (0.86, 1.16)	-559 (-1440, 325)	0.92 (0.79, 1.04)	1.10 (0.90, 1.36)
Ethiopia	2020	154 (-309, 616)	1.03 (0.94, 1.12)	4820 (-68.1, 9700)	1.05 (1.00, 1.10)	0.98 (0.89, 1.09)
	2021	-183 (-744, 378)	0.96 (0.85, 1.08)	6750 (844, 12700)	1.07 (1.01, 1.14)	0.90 (0.78, 1.03)
Kenya	2020	-1520 (-3000, -35.5)	0.76 (0.55, 0.96)	-13800 (-25900, -1700)	0.84 (0.72, 0.97)	0.90 (0.66, 1.23)
	2021	-1380 (-3290, 539)	0.79 (0.54, 1.05)	-12600 (-27600, 2400)	0.86 (0.71, 1.01)	0.92 (0.63, 1.33)
Malawi	2020	-182 (-496, 131)	0.89 (0.70, 1.07)	-785 (-1800, 227)	0.95 (0.88, 1.01)	0.94 (0.75, 1.18)
	2021	-449 (-851, -48)	0.73 (0.53, 0.94)	-1210 (-2410, -3.67)	0.92 (0.84, 1.00)	0.80 (0.60, 1.06)
Mauritius	2020	-5.92 (-9.77, -2.07)	0.46 (0.22, 0.70)	-7.37 (-14.8, 0.0946)	0.94 (0.87, 1.00)	0.49 (0.29, 0.82)
	2021	4.4 (-0.0884, 8.89)	1.42 (0.91, 1.92)	-27.1 (-36, -18.1)	0.76 (0.69, 0.83)	1.86 (1.29, 2.64)
Rwanda	2020	-61.8 (-113, -10.6)	0.87 (0.76, 0.97)	292 (124, 459)	1.05 (1.02, 1.09)	0.82 (0.73, 0.93)
	2021	-140 (-203, -76.6)	0.70 (0.59, 0.82)	75.6 (-127, 278)	1.01 (0.98, 1.05)	0.70 (0.59, 0.82)
Somalia	2020	72.2 (-26, 170)	1.06 (0.98, 1.14)	-1630 (-2840, -431)	0.91 (0.84, 0.97)	1.17 (1.05, 1.30)
	2021	217 (97.5, 336)	1.18 (1.07, 1.29)	-2290 (-3880, -704)	0.87 (0.79, 0.96)	1.35 (1.19, 1.53)
United Republic of Tanzania	2020	482 (-418, 1380)	1.04 (0.96, 1.13)	5080 (884, 9270)	1.07 (1.01, 1.13)	0.98 (0.89, 1.07)
	2021	307 (-948, 1560)	1.02 (0.92, 1.13)	2140 (-3340, 7620)	1.03 (0.96, 1.10)	1.00 (0.89, 1.12)
Uganda	2020			-13000 (-20500, -5530)	0.80 (0.69, 0.90)	
	2021			-17900 (-28600, -7150)	0.75 (0.62, 0.88)	
Zambia	2020	1330 (1030, 1630)	1.70 (1.49, 1.90)	4170 (1860, 6470)	1.13 (1.05, 1.20)	1.50 (1.32, 1.71)

	2021	3020 (2650, 3380)	2.57 (2.27, 2.87)	13300 (10600, 16000)	1.42 (1.32, 1.52)	1.81 (1.56, 2.05)
Southern Sub-Saharan Africa	2020	-8760 (-10000, -7520)	0.42 (0.37, 0.47)	21100 (3690, 38500)	1.10 (1.01, 1.18)	0.38 (0.33, 0.45)
	2021	-1900 (-3360, -446)	0.87 (0.78, 0.96)	8070 (-11100, 27200)	1.04 (0.94, 1.14)	0.84 (0.72, 0.97)
Botswana	2020	-89.7 (-135, -43.9)	0.71 (0.59, 0.84)	-466 (-601, -332)	0.81 (0.76, 0.86)	0.88 (0.74, 1.06)
	2021	-40.8 (-91.4, 9.81)	0.86 (0.70, 1.02)	177 (37.7, 316)	1.08 (1.01, 1.15)	0.79 (0.66, 0.95)
Lesotho	2020	-332 (-529, -134)	0.70 (0.55, 0.85)	-1530 (-2150, -922)	0.72 (0.63, 0.82)	0.96 (0.75, 1.26)
	2021	-369 (-625, -114)	0.68 (0.50, 0.86)	-1350 (-2040, -651)	0.74 (0.63, 0.86)	0.92 (0.67, 1.26)
Namibia	2020	-78.5 (-165, 8.02)	0.85 (0.69, 1.00)	-822 (-1430, -217)	0.89 (0.81, 0.97)	0.95 (0.77, 1.16)
	2021	-25.1 (-125, 75)	0.95 (0.75, 1.15)	-519 (-1230, 189)	0.93 (0.83, 1.02)	1.02 (0.80, 1.31)
South Africa	2020	-7570 (-8750, -6380)	0.33 (0.27, 0.39)	27800 (10500, 45200)	1.16 (1.05, 1.26)	0.28 (0.23, 0.35)
	2021	-1320 (-2710, 59.5)	0.88 (0.76, 1.00)	12400 (-6700, 31500)	1.08 (0.95, 1.20)	0.82 (0.69, 0.96)
Eswatini	2020	-34.1 (-43.7, -24.6)	0.69 (0.62, 0.76)	137 (-113, 387)	1.06 (0.94, 1.19)	0.65 (0.55, 0.75)
	2021	43.5 (34, 52.9)	1.48 (1.35, 1.60)	149 (-106, 404)	1.08 (0.94, 1.23)	1.36 (1.17, 1.61)
Zimbabwe	2020	-664 (-971, -357)	0.63 (0.49, 0.77)	-4070 (-5210, -2930)	0.80 (0.74, 0.85)	0.79 (0.64, 0.98)
	2021	-186 (-543, 170)	0.89 (0.70, 1.09)	-2780 (-4070, -1490)	0.85 (0.79, 0.92)	1.05 (0.84, 1.30)
Western Sub-Saharan Africa	2020	747 (-751, 2240)	1.04 (0.96, 1.11)	1140 (-11900, 14200)	1.00 (0.95, 1.06)	1.03 (0.95, 1.12)
	2021	6290 (4300, 8280)	1.28 (1.18, 1.38)	63400 (46800, 79900)	1.25 (1.17, 1.32)	1.03 (0.93, 1.14)
Benin	2020	-48.9 (-123, 25)	0.84 (0.61, 1.06)	-484 (-995, 26.7)	0.88 (0.77, 1.00)	0.95 (0.69, 1.25)
	2021	-94 (-191, 3.29)	0.71 (0.46, 0.96)	-841 (-1490, -196)	0.81 (0.67, 0.94)	0.88 (0.60, 1.29)
Burkina Faso	2020	-120 (-319, 79.6)	0.81 (0.52, 1.10)	-540 (-1880, 803)	0.91 (0.68, 1.13)	0.89 (0.58, 1.40)
	2021	-44.8 (-310, 221)	0.93 (0.56, 1.31)	91.5 (-1670, 1850)	1.01 (0.73, 1.30)	0.92 (0.57, 1.49)
Cameroon	2020	-121 (-266, 24.1)	0.93 (0.84, 1.01)	-1010 (-2750, 726)	0.95 (0.87, 1.03)	0.97 (0.86, 1.10)
	2021	-151 (-353, 51.6)	0.91 (0.80, 1.03)	-159 (-2420, 2110)	0.99 (0.89, 1.10)	0.92 (0.78, 1.08)
Cabo Verde	2020	9.5 (6.69, 12.3)	2.12 (1.64, 2.60)	-20.3 (-80.4, 39.8)	0.90 (0.63, 1.18)	2.34 (1.65, 3.47)
	2021	6.08 (2.96, 9.21)	1.77 (1.24, 2.29)	-41.4 (-114, 31.1)	0.80 (0.49, 1.11)	2.20 (1.41, 3.55)
Chad	2020	6.96 (-75.3, 89.2)	1.01 (0.91, 1.11)	-615 (-1910, 679)	0.95 (0.85, 1.05)	1.06 (0.92, 1.21)
	2021	10.9 (-90.3, 112)	1.01 (0.89, 1.13)	-159 (-1770, 1450)	0.99 (0.86, 1.11)	1.03 (0.86, 1.22)
Côte d'Ivoire	2020	-295 (-679, 88.3)	0.81 (0.58, 1.03)	-4150 (-9170, 878)	0.82 (0.62, 1.02)	0.99 (0.69, 1.42)
	2021	-297 (-809, 215)	0.82 (0.55, 1.10)	-4480 (-10900, 1930)	0.81 (0.57, 1.05)	1.01 (0.63, 1.55)
Ghana	2020	-380 (-468, -292)	0.78 (0.74, 0.83)	-1130 (-1670, -579)	0.91 (0.87, 0.95)	0.86 (0.80, 0.93)
	2021	-193 (-299, -87.8)	0.89 (0.83, 0.95)	-434 (-1080, 217)	0.96 (0.91, 1.02)	0.92 (0.85, 1.00)
Guinea	2020	66.4 (-368, 500)	1.07 (0.61, 1.53)	-1120 (-2040, -198)	0.93 (0.87, 0.99)	1.15 (0.76, 1.74)
	2021	239 (-318, 797)	1.23 (0.63, 1.84)	808 (-380, 2000)	1.05 (0.98, 1.12)	1.18 (0.73, 1.92)
Guinea-Bissau	2020	-14.2 (-28.7, 0.358)	0.85 (0.71, 0.99)	275 (44.7, 504)	1.13 (1.01, 1.24)	0.76 (0.63, 0.92)
	2021	43.3 (25.9, 60.7)	1.46 (1.23, 1.68)	145 (-135, 425)	1.07 (0.93, 1.20)	1.37 (1.12, 1.63)
Mali	2020	-159 (-440, 122)	0.83 (0.56, 1.10)	-1350 (-2990, 299)	0.82 (0.62, 1.02)	1.01 (0.68, 1.48)
	2021	-230 (-618, 158)	0.78 (0.46, 1.11)	-1760 (-3960, 439)	0.79 (0.55, 1.02)	1.00 (0.60, 1.66)
Mauritania	2020	-10.8 (-42.8, 21.2)	0.96 (0.85, 1.07)	-276 (-515, -37.2)	0.88 (0.79, 0.98)	1.09 (0.93, 1.27)
	2021	26.4 (-13, 65.8)	1.09 (0.95, 1.24)	-242 (-535, 50.7)	0.90 (0.78, 1.01)	1.22 (1.01, 1.47)
Niger	2020	-143 (-283, -3.08)	0.90 (0.80, 0.99)	-1380 (-2850, 93.9)	0.88 (0.76, 1.00)	1.02 (0.86, 1.22)
	2021	-293 (-487, -99.8)	0.81 (0.70, 0.93)	-832 (-2760, 1090)	0.93 (0.78, 1.08)	0.87 (0.70, 1.08)

Nigeria	2020	2440 (1230, 3650)	1.28 (1.12, 1.43)	19900 (9240, 30600)	1.19 (1.08, 1.30)	1.07 (0.92, 1.26)
	2021	7970 (6430, 9520)	1.86 (1.63, 2.09)	80000 (66800, 93300)	1.75 (1.58, 1.91)	1.07 (0.92, 1.24)
Sao Tome and Principe	2020	-3.74 (-9.4, 1.92)	0.68 (0.28, 1.08)	-51.3 (-77.4, -25.1)	0.62 (0.47, 0.77)	1.10 (0.58, 2.01)
	2021	-5.02 (-11.4, 1.32)	0.54 (0.12, 0.97)	-57.3 (-88.3, -26.4)	0.57 (0.39, 0.75)	0.95 (0.43, 2.13)
Sierra Leone	2020	-428 (-940, 83.1)	0.69 (0.38, 1.00)	-6810 (-10900, -2690)	0.68 (0.53, 0.84)	1.01 (0.62, 1.66)
	2021	-667 (-1480, 143)	0.62 (0.25, 0.98)	-8700 (-14600, -2770)	0.65 (0.46, 0.84)	0.95 (0.50, 1.76)
Togo	2020	-54.5 (-93.6, -15.4)	0.75 (0.59, 0.90)	-134 (-281, 13.3)	0.94 (0.88, 1.00)	0.79 (0.63, 1.00)
	2021	-34 (-83, 15)	0.85 (0.65, 1.05)	-23.1 (-198, 152)	0.99 (0.91, 1.07)	0.86 (0.67, 1.10)
South Sudan	2020	-588 (-1070, -110)	0.57 (0.31, 0.83)	-9770 (-17200, -2380)	0.60 (0.37, 0.84)	0.94 (0.51, 1.71)
	2021	-1050 (-1970, -132)	0.48 (0.16, 0.79)	-19400 (-33300, -5610)	0.46 (0.20, 0.72)	1.05 (0.44, 2.48)

Table S3. Bivariate linear regression results for observed to expected ratios of tuberculosis diagnoses during the COVID-19 pandemic in 2020

		Coefficient (95% CI)	Standardized coefficient (95% CI)	Pearson *r* (95% CI)
**Tuberculosis risk factors**	Latent TB prevalence (per 1%)	1.002 (1.000, 1.005)	0.022 (-0.004, 0.048)	0.130 (-0.022, 0.275)
	Indoor air pollution prevalence (per 5%)	1.007 (1.003, 1.010)	0.046 (0.022, 0.071)	0.275 (0.129, 0.409)
	Population density (per 5% population living over 1000 ppl/sqkm)	0.997 (0.991, 1.003)	-0.012 (-0.038, 0.013)	-0.073 (-0.222, 0.079)
	Age-standardized HIV prevalence rate (per 1,000)	1.000 (1.000, 1.001)	0.015 (-0.010, 0.041)	0.091 (-0.061, 0.238)
	Age-standardized smoking prevalence (per 5%)	0.974 (0.959, 0.989)	-0.043 (-0.068, -0.018)	-0.252 (-0.389, -0.106)
	Liters of alcohol consumed per capita	0.993 (0.987, 0.999)	-0.031 (-0.057, -0.006)	-0.183 (-0.325, -0.033)
	Age-standardized diabetes prevalence rate (per 1,000)	1.000 (0.999, 1.001)	-0.009 (-0.035, 0.017)	-0.055 (-0.204, 0.097)
**Health system factors**	Healthcare access and quality index (per 5 score)	0.991 (0.985, 0.996)	-0.043 (-0.068, -0.017)	-0.251 (-0.387, -0.104)
	Health worker density (health workers per 1,000)	0.999 (0.997, 1.001)	-0.015 (-0.041, 0.010)	-0.091 (-0.239, 0.060)
	Hospital Beds (per 1000)	0.989 (0.979, 1.000)	-0.026 (-0.051, -0.000)	-0.151 (-0.296, -0.000)
	Pandemic preparedness (per 5 score in GHS Index)	0.988 (0.979, 0.997)	-0.034 (-0.059, -0.008)	-0.198 (-0.338, -0.048)
**Socio-demographic factors**	Lag distributed income per capita (per 25%)	0.995 (0.990, 0.999)	-0.029 (-0.054, -0.003)	-0.168 (-0.311, -0.018)
	Percent of population 65 and above (per 5%)	0.965 (0.947, 0.984)	-0.046 (-0.071, -0.021)	-0.268 (-0.403, -0.122)
	Education (2 years per capita)	0.968 (0.951, 0.985)	-0.047 (-0.072, -0.022)	-0.278 (-0.412, -0.133)
	Socio-demographic index (per 5 score)	0.988 (0.981, 0.995)	-0.042 (-0.067, -0.017)	-0.248 (-0.384, -0.100)
**Public health and social measures**	Stringency index (per 5 score)	0.988 (0.981, 0.996)	-0.040 (-0.066, -0.015)	-0.245 (-0.386, -0.093)
	Stay-at-home order (per 30 days in place)	0.987 (0.980, 0.995)	-0.042 (-0.067, -0.017)	-0.254 (-0.394, -0.102)
	School closure (per 30 days in place)	0.981 (0.972, 0.990)	-0.051 (-0.076, -0.026)	-0.308 (-0.443, -0.161)
	Workplace closures (per 30 days in place)	0.989 (0.981, 0.997)	-0.034 (-0.059, -0.008)	-0.204 (-0.349, -0.050)
	Internal movement restrictions (per 30 days in place)	0.994 (0.986, 1.002)	-0.019 (-0.045, 0.007)	-0.114 (-0.265, 0.042)
	Mask use prevalence (per 5%)	0.992 (0.985, 1.000)	-0.026 (-0.051, -0.000)	-0.154 (-0.298, -0.003)
	Mobility (per 5%)	1.021 (1.008, 1.035)	0.040 (0.015, 0.066)	0.239 (0.092, 0.377)
**COVID-19 factors**	Reported COVID-19 case rate (per 1,000)	0.997 (0.996, 0.998)	-0.054 (-0.078, -0.029)	-0.318 (-0.448, -0.175)
	SARS-CoV-2 infection rate (per 25%)	0.994 (0.990, 0.999)	-0.034 (-0.059, -0.009)	-0.202 (-0.342, -0.052)

	Age-standardized reported COVID-19 death rate (per 1,000)	0.938 (0.904, 0.973)	-0.043 (-0.068, -0.018)	-0.255 (-0.391, -0.108)
	Age-standardized total COVID-19 death rate (per 1,000)	1.007 (0.993, 1.022)	0.013 (-0.013, 0.039)	0.077 (-0.075, 0.225)
	Age-standardized excess death rate (per 1,000)	1.006 (0.995, 1.018)	0.014 (-0.011, 0.040)	0.086 (-0.066, 0.234)

Table S4. Bivariate linear regression results for observed to expected ratios of tuberculosis diagnoses during the COVID-19 pandemic in 2021

		**Coefficient (95% CI)**	**Standardized coefficient (95% CI)**	**Pearson *r* (95% CI)**
**Tuberculosis risk factors**	Latent TB prevalence (per 1%)	1.000 (0.997, 1.003)	-0.001 (-0.032, 0.029)	-0.006 (-0.157, 0.144)
	Indoor air pollution prevalence (per 5%)	1.006 (1.002, 1.010)	0.043 (0.013, 0.073)	0.214 (0.065, 0.353)
	Population density (per 5% population living over 1000 ppl/sqkm)	0.999 (0.991, 1.006)	-0.005 (-0.036, 0.025)	-0.027 (-0.177, 0.124)
	Age-standardized HIV prevalence rate (per 1,000)	1.001 (1.000, 1.002)	0.026 (-0.004, 0.056)	0.130 (-0.021, 0.275)
	Age-standardized smoking prevalence (per 5%)	0.957 (0.940, 0.974)	-0.071 (-0.100, -0.043)	-0.355 (-0.480, -0.216)
	Liters of alcohol consumed per capita	0.996 (0.989, 1.003)	-0.019 (-0.050, 0.011)	-0.096 (-0.243, 0.055)
	Age-standardized diabetes prevalence rate (per 1,000)	0.999 (0.998, 1.000)	-0.035 (-0.065, -0.005)	-0.174 (-0.316, -0.024)
**Health system factors**	Healthcare access and quality index (per 5 score)	0.991 (0.985, 0.998)	-0.040 (-0.070, -0.010)	-0.197 (-0.337, -0.048)
	Health worker density (health workers per 1,000)	1.000 (0.997, 1.002)	-0.005 (-0.036, 0.026)	-0.025 (-0.175, 0.126)
	Hospital Beds (per 1000)	0.987 (0.974, 0.999)	-0.032 (-0.062, -0.002)	-0.158 (-0.301, -0.008)
	Pandemic preparedness (per 5 score in GHS Index)	0.990 (0.979, 1.001)	-0.028 (-0.058, 0.002)	-0.138 (-0.283, 0.012)
**Socio-demographic factors**	Lag distributed income per capita (per 25%)	0.995 (0.990, 1.000)	-0.028 (-0.058, 0.002)	-0.140 (-0.285, 0.011)
	Percent of population 65 and above (per 5%)	0.966 (0.944, 0.988)	-0.045 (-0.075, -0.016)	-0.226 (-0.364, -0.078)
	Education (2 years per capita)	0.973 (0.953, 0.994)	-0.039 (-0.069, -0.009)	-0.194 (-0.335, -0.045)
	Socio-demographic index (per 5 score)	0.988 (0.980, 0.996)	-0.043 (-0.073, -0.013)	-0.216 (-0.355, -0.067)
**Public health and social measures**	Stringency index (per 5 score)	0.985 (0.976, 0.994)	-0.048 (-0.077, -0.018)	-0.249 (-0.390, -0.098)
	Stay-at-home order (per 30 days in place)	0.996 (0.992, 1.000)	-0.028 (-0.058, 0.002)	-0.147 (-0.295, 0.008)
	School closure (per 30 days in place)	0.991 (0.986, 0.996)	-0.049 (-0.078, -0.020)	-0.259 (-0.398, -0.108)
	Workplace closures (per 30 days in place)	0.994 (0.989, 0.999)	-0.037 (-0.066, -0.008)	-0.195 (-0.340, -0.041)
	Internal movement restrictions (per 30 days in place)	0.998 (0.994, 1.003)	-0.011 (-0.041, 0.018)	-0.060 (-0.213, 0.096)
	Mask use prevalence (per 5%)	0.995 (0.987, 1.003)	-0.019 (-0.050, 0.011)	-0.096 (-0.243, 0.055)
	Mobility (per 5%)	1.013 (1.003, 1.022)	0.041 (0.011, 0.071)	0.203 (0.054, 0.343)
**COVID-19 factors**	Reported COVID-19 case rate (per 1,000)	0.999 (0.999, 1.000)	-0.041 (-0.071, -0.011)	-0.205 (-0.346, -0.056)
	SARS-CoV-2 infection rate (per 25%)	1.003 (0.995, 1.010)	0.011 (-0.020, 0.041)	0.054 (-0.097, 0.203)

	Age-standardized reported COVID-19 death rate (per 1,000)	0.967 (0.943, 0.993)	-0.039 (-0.069, -0.009)	-0.193 (-0.334, -0.044)
	Age-standardized total COVID-19 death rate (per 1,000)	1.011 (1.000, 1.021)	0.030 (-0.001, 0.060)	0.148 (-0.003, 0.292)
	Age-standardized excess death rate (per 1,000)	1.008 (1.001, 1.016)	0.033 (0.002, 0.063)	0.162 (0.012, 0.305)
